# Complex magnetic properties associated with competing local and itinerant magnetism in ${\text {Pr}}_2 {\text {Co}}_{0.86} {\text {Si}}_{2.88}$

**DOI:** 10.1038/s41598-021-90751-0

**Published:** 2021-06-24

**Authors:** Mily Kundu, Santanu Pakhira, Renu Choudhary, Durga Paudyal, N. Lakshminarasimhan, Maxim Avdeev, Stephen Cottrell, Devashibhai Adroja, R. Ranganathan, Chandan Mazumdar

**Affiliations:** 1 Condensed Matter Physics Division, Saha Institute of Nuclear Physics, 1/AF Bidhannagar, Kolkata, 700064 India; 2 Ames Laboratory-USDOE, Iowa State University, Ames, Iowa 50011 USA; 3 Electrical and Computer Engineering Department, Iowa State University, Ames, Iowa 50011 USA; 4 Electro-organic and Materials Electrochemistry Division, CSIR-Central Electrochemical Research Institute, Karaikudi, 630 003 India; 5 Academy of Scientific and Innovative Research (AcSIR), Ghaziabad, 201 002 India; 6 Australian Nuclear Science and Technology Organisation (ANSTO), New Illawarra Road, Lucas Heights, NSW 2234 Australia; 7 School of Chemistry, The University of Sydney, Sydney, NSW 2006 Australia; 8 ISIS Facility, STFC, Rutherford Appleton Laboratory, Chilton, Didcot, Oxfordshire OX11 0QX UK; 9 Highly Correlated Matter Research Group, Physics Department, University of Johannesburg, PO Box 524, Auckland Park, 2006 South Africa

**Keywords:** Magnetic properties and materials, Magnetic properties and materials

## Abstract

Ternary intermetallic compound ${\text {Pr}}_2 {\text {Co}}_{0.86} {\text {Si}}_{2.88}$ has been synthesized in single phase and characterized by x-ray diffraction, scanning electron microscopy with energy dispersive x-ray spectroscopy (SEM-EDX) analysis, magnetization, heat capacity, neutron diffraction and muon spin rotation/relaxation ($\mu$SR) measurements. The polycrystalline compound was synthesized in single phase by introducing necessary vacancies in Co/Si sites. Magnetic, heat capacity, and zero-field neutron diffraction studies reveal that the system undergoes magnetic transition below $\sim$4 K. Neutron diffraction measurement further reveals that the magnetic ordering is antiferromagnetic in nature with an weak ordered moment. The high temperature magnetic phase has been attributed to glassy in nature consisting of ferromagnetic clusters of itinerant (3*d*) Co moments as evident by the development of internal field in zero-field $\mu$SR below 50 K. The density-functional theory (DFT) calculations suggest that the low temperature magnetic transition is associated with antiferromagnetic coupling between Pr 4*f* and Co 3*d* spins. Pr moments show spin fluctuation along with unconventional orbital moment quenching due to crystal field. The evolution of the symmetry and the crystalline electric field environment of Pr-ions are also studied and compared theoretically between the elemental Pr and when it is coupled with other elements such as Co. The localized moment of Pr 4*f* and itinerant moment of Co 3*d* compete with each other below $\sim$20 K resulting in an unusual temperature dependence of magnetic coercivity in the system.

## Introduction

In the last few decades, systems with competing magnetic interactions are extensively studied due to their interesting ground state properties^1–10^. Since these systems are unable to minimize all the interaction energies simultaneously, one may often expect the occurrence of multiple magnetic ground states^11^. There exist a wide range of sources for a system to exhibit such competing interactions, e.g.﻿ simultaneous presence of ferromagnetic (FM) and antiferromagnetic (AFM) interactions, geometric frustration, etc﻿. The presence of atomic/magnetic disorder are also known to influence the local magnetic interaction in a significant manner. In the situation when both frustration and disorder are present, the system may exhibit spin/cluster glass behaviour^12,13^.

Recently the discovery of field-induced skyrmionic phase in isostructural frustrated compound ${\text {Gd}}_2 {\text {PdSi}}_3$^14^ have sparked a new interest to the community in order to find the exact role of magnetic frustration on the formation of such topological spin textures. It is worth mentioning here that, the skyrmion phase in ${\text {Gd}}_2 {\text {PdSi}}_3$ is formed by localized spins of Gd ions as the magnetic contribution from Pd atom is negligible. Beside ${\text {Gd}}_2 {\text {PdSi}}_3$, many other ternary ${\text {R}}_2 {\text {TX}}_3$ (where R = rare-earth, T = transition metal, X = Si, Ge, In) type of intermetallic compounds have also been reported to exhibit competing magnetic interactions due to their lattice geometry, presence of both FM and AFM type interactions, and incorporation of site/bond disorders in their structure^1,6,15,16^. Due to the complex magnetic ground states, those often exhibit a wide range of interesting physical properties viz﻿. spin-glass behavior, magnetic memory effect, Kondo like behaviour and multiple magnetic transitions^1,17–19^. Most of these compounds crystallize in the hexagonal ${\text {AlB}}_2$- type structure in space group *P6/mmm*. In this structure, the R-ions resides at the Al sites (Wyckoff position: 1*a*) forming a hexagonal structure, while the T and X ions are randomly distributed (Wyckoff position: 2*d*) in a plane sandwiched between two hexagonal layers of R-ions^2,18^. The exchange interaction among the R-ions is of Ruderman–Kittel–Kasuya–Yosida (RKKY) type and if the interaction is antiferromagnetic in nature, geometry may fecilitate magnetic frustration among the R-moments. The distance between nearest-neighbour (NN) and next-nearest-neighbour (NNN) R-ions are comparable in ${\text {R}}_2 {\text {TX}}_3$ type of compounds, that in turn can trigger magnetic frustration in those systems. In addition to magnetic frustration, random distribution of T and X ions (sandwiched between two hexagonal layers of R ions) causes a local variation in electronic environment between the R ions that may induce different magnetic interaction and coexisting magnetically different phases in a crystallographically single phase compound^18^. As a result, for a particular set of T and X, compounds with different rare-earths may even exhibit magnetically different properties. For example, the series of compounds such as ${\text {Gd}}_2 {\text {NiSi}}_3$ and ${\text {Er}}_2 {\text {NiSi}}_3$ (forming in stoichiometric proportion) exhibit long range magnetic ordering that coexists with frustrated glassy components^18^. On the other hand, compounds with other R elements form in defect structure where the volume fraction of spin/cluster glass component enhances considerably at the expense of long range ordered phase^1,17^. Even a slight variation in composition are known to alter the magnetic property significantly as seen in the ac susceptibility data of polycrystalline ${\text {Pr}}_2 {\text {Ni}}_{0.95} {\text {Si}}_{2.95}$ that exhibits an additional peak showing a reverse frequency dependence^3^ in contrast to that generally observed in a spin-glass system, but is absent in single crystalline full stoichiometric ${\text {Pr}}_2 {\text {NiSi}}_3$^20^.

**Figure 1:**
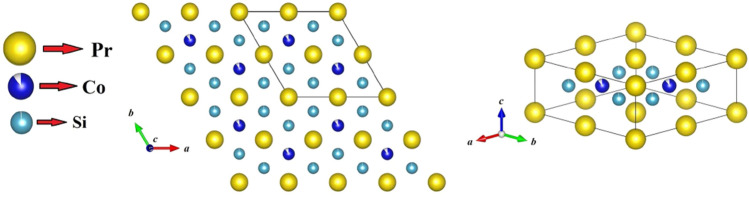
Crystal structure of ${\text {Pr}}_2 {\text {Co}}_{0.86} {\text {Si}}_{2.88}$ belonging to the ${\text {U}}_2 {\text {RuSi}}_3$-type structure (space group: *P6/mmm*) that is considered as a superstructure of ${\text {AlB}}_2$-type structure. The doubling of the “*a*” (and “*b*”) lattice parameters in the superstructure allows splitting of Wyckoff sites and a regular arrangement of Co and Si atoms, which otherwise would not be possible in the ${\text {AlB}}_2$-type structure. The white sectors in the Co and Si atom symbols show the proportion of the site-vacancies as estimated from the XRD data. Unit cell is marked in the left panel.

In such a magnetically complex system, one may have an additional magnetic interaction, by introducing a moment carrying element in the transition metal site. Although the magnetic moment contribution of T-atoms in most reported members of ${\text {R}}_2 {\text {TSi}}_3$ are found to be negligible as compared to the R contribution, a detectable 3*d*-moment associated with Co-atom has been reported in ${\text {Ce}}_2 {\text {CoSi}}_3$^21^. The presence of the Co-moment could be an additional source of magnetic interaction that may allow us to investigate any complex phenomena associated with competing magnetic interactions between localized (4*f*) and local/itinerant (3*d*) moment in ${\text {R}}_2 {\text {CoSi}}_3$ series of compounds. These Co-based compounds, however have been reported to crystallize in a superstructure form (${\text {U}}_2 {\text {RuSi}}_3$-type) of the basic ${\text {AlB}}_2$-type crystal structure, where the Co and Si atoms are placed in a regular order having separate Wyckoff positions^21^. To accommodate such ordered arrangement of Co/Si atoms, the lattice parameters of the ${\text {U}}_2 {\text {RuSi}}_3$-type structure exhibit a two-fold increase along the *a*-axis, although the space group (*P6/mmm*) remains invariant (Fig. 1). Even the Ir-based analogue, ${\text {Ce}}_2 {\text {IrSi}}_3$ has also been reported to form in the same superstructure type with same space group^16^. In this work, we report the synthesis of a new polycrystalline compound ${\text {Pr}}_2 {\text {Co}}_{0.86} {\text {Si}}_{2.88}$ and characterize by means of x-ray diffraction (XRD), SEM-EDX, magnetic, heat capacity, neutron diffraction (ND) and muon spin relaxation ($\mu$SR) measurements. A complementary theoretical analysis has also been carried out to understand the magnetic ground state of this compound. The presence of both local and itinerant magnetism appear to lead the system towards a complex magnetic ground state. Most interestingly, as a consequence of the competing magnetic interactions, we have observed an anomalous temperature dependence of magnetic coercivity in the system—a phenomenon hitherto rarely emphasized. To understand the origin of this unusual anomaly, we have utilized the above mentioned experimental techniques as well as theoretical calculations and present our results depicting a competing local and itinerant moment picture.

**Figure 2:**
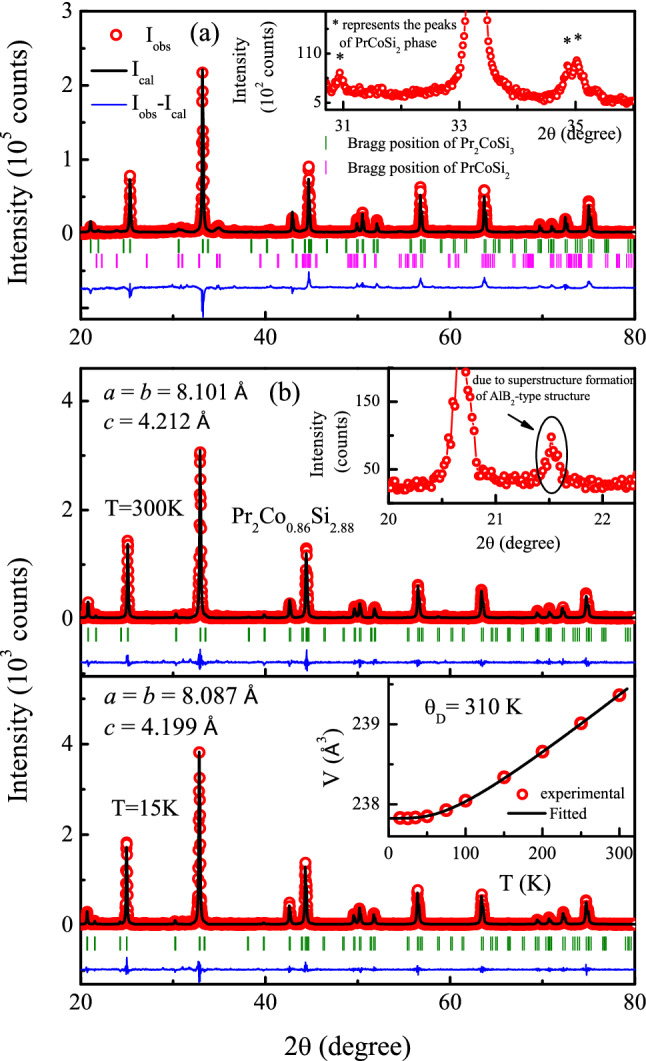
**(a)** Room temperature XRD pattern of ${\text {Pr}}_2 {\text {CoSi}}_3$. Inset of **(a)** shows the presence of secondary phase ${\text {PrCoSi}}_2$ in ${\text {Pr}}_2 {\text {CoSi}}_3$. **(b)** The XRD patterns of ${\text {Pr}}_2 {\text {Co}}_{0.86} {\text {Si}}_{2.88}$ at $T = 300 \, \text {K}$ (top) and $T = 15\, \text {K}$ (bottom) along with full-Rietveld analysis. Inset of (top) presents the major peak associated with the superstructure of ${\text {AlB}}_2$-type structure. Inset of (bottom) presents the temperature dependence of unit-cell volume along with fit using Eq. (1). Estimated errors are smaller than the symbol size.

**Figure 3:**
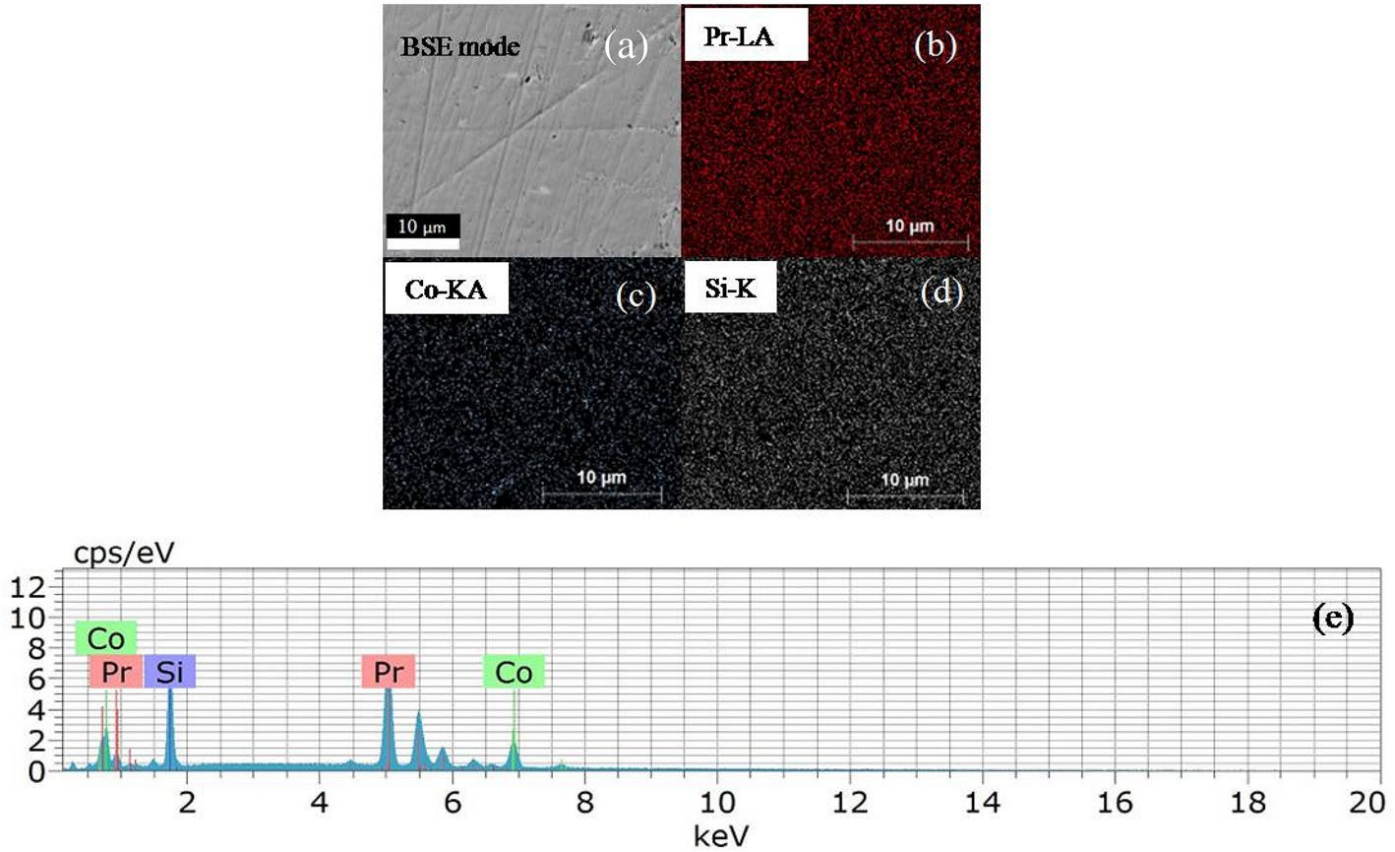
**(a)** Backscattered electron (BSE) image and **(b–d)** elemental mapping by SEM equipped with EDX on the surface of ${\text {Pr}}_2 {\text {Co}}_{0.86} {\text {Si}}_{2.88}$ from the region presented in **(a)**. **(e)** presents EDX results of ${\text {Pr}}_2 {\text {Co}}_{0.86} {\text {Si}}_{2.88}$.

**Table 1:** Crystallographic parameters and composition from XRD and SEM-EDX analysis at room-temperature.

Compound	${\text {Pr}}_2 {\text {Co}}_{0.86} {\text {Si}}_{2.88}$					
Structure	${\text {U}}_2 {\text {RuSi}}_3$ type					
Space group	*P6/mmm*					
**Lattice parameters**						
*a* (Å)	8.101					
*c* (Å)	4.212					
${\text {R}}_{\rm{f}}$(%)	0.766					
${\text {R}}_{{\rm{Bragg}}}$(%)	1.33					

Atomic parameters					Estimated composition	
Atom	Wyckoff site	x	y	z	Full-Rietveld	EDX
Pr1	1*a*	0	0	0		
Pr2	3*f*	1/2	0	0		
Co	2*d*	1/3	2/3	1/2	${\text {Pr}}_2 {\text {Co}}_{0.95(1)} {\text {Si}}_{2.96(2)}$	${\text {Pr}}_2 {\text {Co}}_{0.98(1)} {\text {Si}}_{2.87(1)}$
Si	6*m*	0.1671(1)	0.3342(2)	1/2		

## Results and discussions

### Structural details and phase analysis

The XRD pattern of Pr–Co–Si composition, synthesized in stoichiometric ratio 2 : 1 : 3 for Pr, Co and Si, respectively, is shown in Fig. 2(a). The ${\text {R}}_2 {\text {TX}}_3$ type of compounds are generally known to form primarily in two different hexagonal crystal structures with space groups *P6/mmm*^1,6^ and ${\textit{P6}}_3/{\textit{mmc}}$ (or ${\textit{P}}{\bar{6}}{\textit{2c}}$)^22,23^. It was also reported that the arc furnace synthesis of these ${\text {R}}_2 {\text {TX}}_3$ type of polycrystalline compounds often results in an additional secondary phase, either of ${\text {RT}}_2 {\text {X}}_2$ type^24,25^ or ${\text {RTX}}_2$ type^2^. The major peaks in XRD pattern (at room temperature) of our sample could be indexed by considering the simple ${\text {AlB}}_2$-type crystal structure belonging to *P6/mmm* space group^18^ with $a\approx c$, while a few additional peaks remain unaccounted. Most of these additional peaks remain unindexed, even if we consider the ${\text {U}}_2 {\text {RuSi}}_3$-type crystal structure ($a\approx 2c$) having fully ordered hexagonal ${\text {AlB}}_2$-type structure, employed earlier to describe the crystal structure of ${\text {Ce}}_2 {\text {CoSi}}_3$^21^ and ${\text {Ce}}_2 {\text {IrSi}}_3$^16^. Through full-Rietveld analysis of the XRD pattern, we found that the XRD pattern can be explained well considering the presence of $\sim$14 % (weight fraction) secondary phase as ${\text {PrCoSi}}_2$ (space group *Cmcm*), in addition to the main phase ${\text {Pr}}_2 {\text {CoSi}}_3$ (${\text {U}}_2 {\text {RuSi}}_3$-type structure with space group *P6/mmm*).

Following the established treatment of obtaining single phase in similar compounds^2,26^, we have intentionally synthesized non-stoichiometric compound with starting composition ${\text {Pr}}_2 {\text {Co}}_{0.86} {\text {Si}}_{2.88}$, estimated after subtracting the excess elemental ratio which is responsible for the formation of the above mentioned secondary phase, ${\text {PrCoSi}}_2$. Full-Rietveld analysis of the XRD pattern of this defect composition, ${\text {Pr}}_2 {\text {Co}}_{0.86} {\text {Si}}_{2.88}$, barring only a few peaks (at $2\theta =21.95^{\circ }$, $30.6^{\circ }$) of very weak intensity (${\text {I/I}}_{max}< 5\%$) could be explained by ${\text {AlB}}_2$-type hexagonal crystal structure (space group *P6/mmm*). However, all the peaks, detected within the resolution limit of the measurement, could be indexed through the ${\text {U}}_2 {\text {RuSi}}_3$-type structure (space group *P6/mmm*), suggesting the formation of the compound in essentially single phase (Fig. 2(b)top). In this hexagonal structure, R atoms occupy two different crystallographical sites both of which having hexagonal prismatic coordination: one Pr site is surrounded by 12 Si atoms while the other Pr atom has 4 Co and 8 Si atoms as nearest neighbour. The Co and Si atoms are positioned in such a way that layers of Pr atoms are separated by an intermediate layer containing Co and Si atoms (Fig. 1). The refined composition obtained from the analysis is ${\text {Pr}}_2 {\text {Co}}_{0.95(1)} {\text {Si}}_{2.96(2)}$, which is close to the starting defect composition. The neutron diffraction (ND) experiment (discussed later) yielded similarly close composition ${\text {Pr}}_2 {\text {Co}}_{0.90} {\text {Si}}_{2.96}$. The single phase nature of the sample is also confirmed by SEM image taken in back-scattered electron (BSE) mode (Fig. 3), that further shows an essentially homogeneous character of the material. The analysis of EDX spectrum reveals the composition to be ${\text {Pr}}_2 {\text {Co}}_{0.98(1)} {\text {Si}}_{2.87(1)}$, which is also close to the composition obtained from full-Rietveld analysis of XRD and ND patterns. The crystallographic data and elemental composition obtained from XRD analysis as well as EDX measurements, are listed in Table 1.

An interesting point may be noted here is that although the anisotropy parameter, $a\approx 2c$, the distance between the R atoms along ‘*a*’ and ‘*c*’ direction are almost equal so that nearest-neighbour ($J_{\rm{NN}}$) and next-nearest-neighbour ($J_{\rm{NNN}}$) interactions considering only R to be magnetic, are of comparable strength. A very similar feature had earlier been noted in ${\text {R}}_2 {\text {NiSi}}_3$ system that forms in ${\text {AlB}}_2$-type structure^18^. As the ${\text {U}}_2 {\text {RuSi}}_3$-type structure essentially forms as a superstructure of ${\text {AlB}}_2$-type structure by doubling the *a*-lattice parameter, the average atom-atom distances essentially do not exhibit any significant variation in both the structure-types. In addition to the above mentioned feature of $J_{\rm{NN}}\sim J_{\rm{NNN}}$, if the transition element possesses magnetic moment, then the numbers of exchange parameters, R–R, R–T, and T–T, their sign and strength will be more complex. Such competing exchange interactions may lead to frustration and the system may exhibit spin/cluster glass freezing in the threshold limit of disorder^15,27,28^.

In order to check the evolution of crystal structure at low temperature, we have performed XRD measurements at different temperatures in the range $15 \, \text {K}~\le ~ T~ \le ~300 \, \text {K}$. Analysis of these data of ${\text {Pr}}_2 {\text {Co}}_{0.86} {\text {Si}}_{2.88}$ indicates that the crystal structure remains invariant down to 15 K (lowest measurable temperature in the laboratory), as shown in Fig. 2(b). The temperature dependence of lattice volume of the compound is fitted using the standard thermal contraction rule

1 $$\begin{aligned} V(T)&= \frac{\gamma U(T)}{K_{0}}+V_{0} \end{aligned}$$

Here $V_0$ represents the unit cell volume of the system at $T= 0 \, \text {K}$, $K_{0}$ is bulk modulus, $\gamma$ signifies the Grüneisen parameter and *U(T)* is the internal energy which could be expressed as

2 $$\begin{aligned} U(T)\, = \, & 9nRT{\left( \frac{T}{\theta _{\rm{D}}}\right) }^3\int _{0}^{\theta _{D}/T}\frac{x^3}{e^x-1} dx \end{aligned}$$

where $\theta _{\rm{D}}$ is the Debye temperature and *n* denotes the number of atoms per formula unit (f.u.). The temperature dependence of the lattice volume in ${\text {Pr}}_2 {\text {Co}}_{0.86} {\text {Si}}_{2.88}$ can be fitted well with this Debye approximation, yielding $\theta _{\rm{D}} = 260(4) \, \text {K}$ (Inset of bottom panel in Fig. 2(b)). A similar values of Debye temperatures have earlier been reported in a diverse range of intermetallic^18,29,30^ as well as oxide^31^ compounds.

### Theoretical investigation

To investigate the nature of magnetic ground state and associated magnetic interactions, we have carried out density-functional theory (DFT) calculations for ${\text {Pr}}_2 {\text {CoSi}}_3$. Within, we have employed the generalized gradient approximation (GGA) for the exchange and correlation functional and used the projector augmented wave (PAW) method^32,33^, as implemented in the Vienna Ab-initio Simulation Package (VASP). We have also used spin-orbit coupling, on-site electron correlation (Hubbard U) parameter = 6 eV, and on-site exchange parameter, $J_{ex} = 0.7 \, \text {eV}$ for strongly correlated Pr-4*f* states^34^. We have used the low-temperature ($T = 15 \, \text {K}$) experimental lattice parameters of $a = b = 8.087~{\AA }$ and $c = 4.199~{\AA }$. The convergence criterion for the self-consistent calculations is $10^{-6} \, \text {eV}$ for the total energy per supercell, and an energy cutoff of 330 eV is used for the electronic wave functions. The k-point integration was performed using a tetrahedron method with Bloch corrections^35^. A $\Gamma$-centered grid of $6\times 6 \times 12$ k-points was used for Brillouin zone sampling.

**Figure 4:**
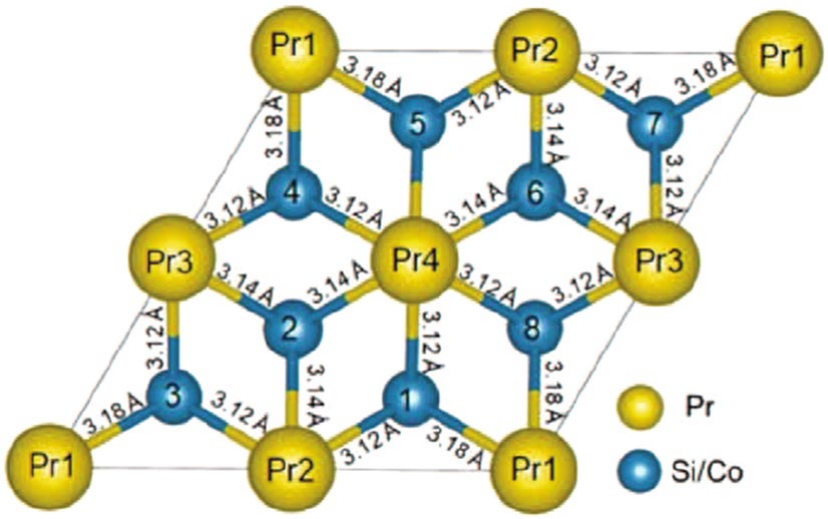
2D-view of ${\text {Pr}}_2 {\text {CoSi}}_3$ structure (${\text {AlB}}_2$-type) showing the positions 1–4 for Pr atoms (yellow) and 1–8 for Si/Co-site (light blue) in configuration I.

**Figure 5:**
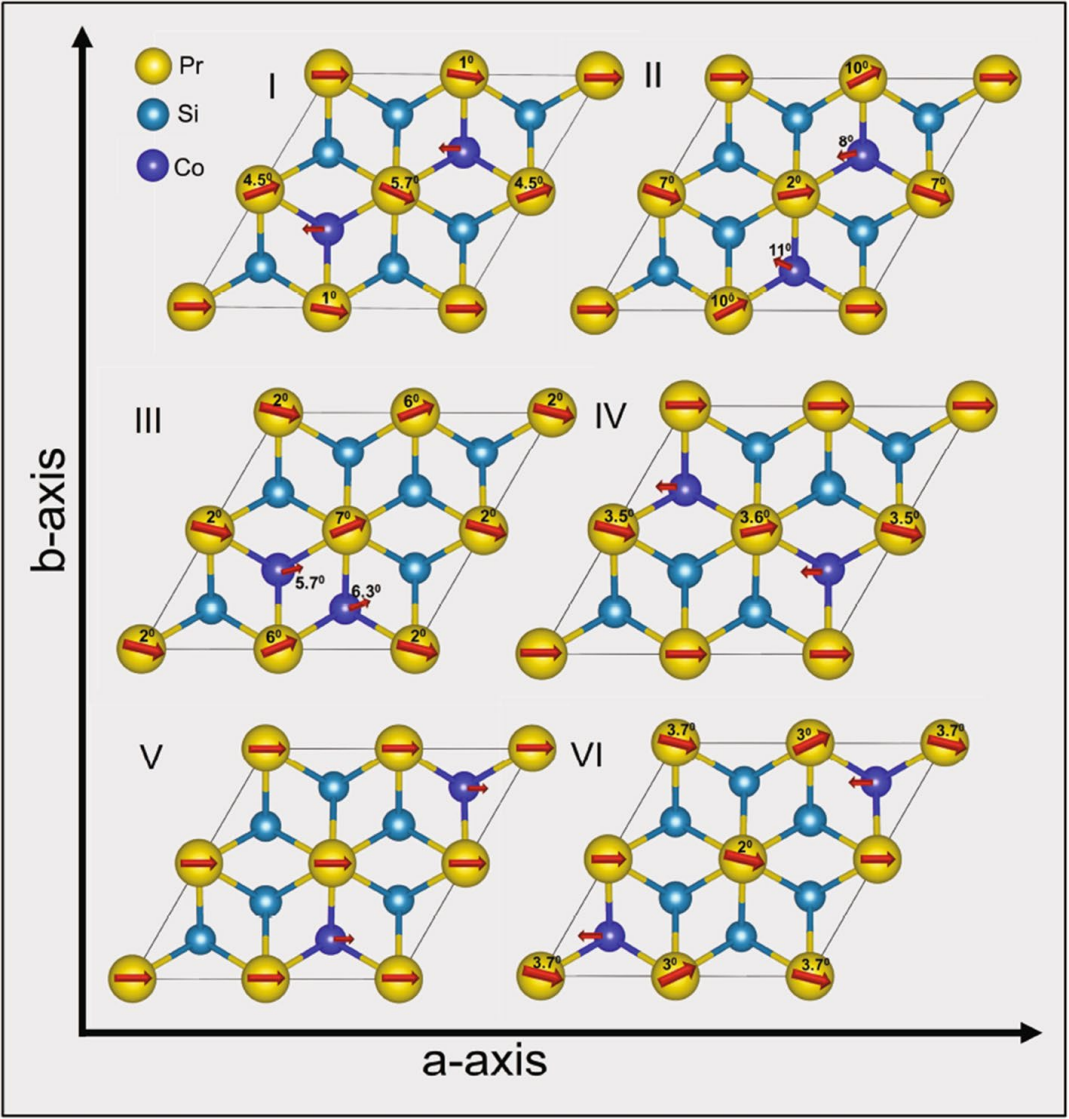
2D-view of ${\text {Pr}}_2 {\text {CoSi}}_3$ structure belonging to ${\text {AlB}}_2$-type crystal structure by considering a $2a \times 2b \times c$ supercell. The angle between *a*- and *b*-axis, as presented in the figure, is $60^{\circ }$. Depending on the relative positions of Co and Si atoms, six different configurations are allowed. The configuration I, belonging to the ${\text {U}}_2 {\text {RuSi}}_3$-type superstructure, is found to be energetically most stable. The spin rotation angles around the *a*-axis are also displayed along with the respective atomic symbols.

To test that the ${\text {U}}_2 {\text {RuSi}}_3$-type structure is the ground state configuration, we started the theoretical calculation by considering the basic ${\text {AlB}}_2$-type crystal structure, and all possible combinations of Si and Co atoms. Out of eight Co/Si positions in ${\text {Pr}}_2 {\text {CoSi}}_3$ (Fig. 4), two are assigned as Co and six are assigned as Si and the systems are categorized from I-VI based on the position of cobalt atoms in the supercell (Fig. 5). From the comparison of ground-state energies of all systems, it is found that system-I, mimicking the ${\text {U}}_2 {\text {RuSi}}_3$-type superstructure, is indeed the energetically most stable configuration at low temperature. The minimum energy configurations for the different systems I-VI have been computed (Table 2) and the corresponding spin structures are presented in Fig. 5. We have checked for both the FM as well as the AFM coupling between Pr-Pr atoms and Pr-Co atoms and it is found that AFM coupling between Pr and Co atoms is energetically most favourable. Our calculation also reveals the presence of a weak indirect exchange interaction between Pr 4*f*-4*f* electrons, as indicated by the estimated small magnitude of the Pr 5*d* magnetic moment^34^. Pr-atoms in all the systems except system-V show spin fluctuation, as the Pr-4*f*’s spin appear to rotate at an angle of 2–10 degree around *a*-axis in *xy*-plane. In systems I, II, IV, and VI, Co spin aligns antiparallel to Pr-spin with magnetic moment of $\sim$0.18 $\mu _B$ per Co-atom whereas in systems III and V, Co-spin is parallel to the Pr-spin with magnetic moment of 0.54 and 0.47 $\mu _B$ per Co-atom, respectively. Pr-orbital ($\sim$2.3-4.2 $\mu _B$) moment found to be antiparallel to Pr-spin ($\sim$1.9-2.0 $\mu _B$) moment i.e. $J=L-S$, which is expected in light R elements from the Hund’s rule, although Pr’s orbital moment gets partially quenched in this structure due to the crystalline electric field effect. Interestingly, even the quenching of orbital moment appear to be strongly depending on its position in the crystal, i.e.﻿, on the nearest-neighbour environment. The orbital moment is highly quenched on the Pr-atoms nearest to Co-atom and less quenched on the Pr-atom which is second nearest-neighbor of Co-atom (see orbital moment of all Pr atoms in Table 3). The proximity effect is quite evident in these systems as the spin-fluctuation behaviour appear to be more prominent in those Pr-atoms which are close to the Co-atoms. Thus, the varying competition between Co’s moment with Pr’s moment leads to spin fluctuations of Pr and also resulting in different magnetic moment among different Pr ions belonging to the same crystal structure.

**Table 2:** Table defining the system I–VI on the basis of cobalt’s position with corresponding ground state energies (GSE).

Configuration	Co’s position	GSE eV per f.u.
System I	2 & 6	− 40.3184
System II	1 & 6	− 39.876
System III	1 & 2	− 39.2531
System IV	4 & 8	− 40.1522
System V	1 & 7	− 38.9657
System VI	3 & 7	− 40.1409

The information of magnetic moment of all the systems are summarised in Table 3. System I, representing the most stable structure of ${\text {Pr}}_2 {\text {CoSi}}_3$, has a total magnetic moment of $1.69 \, \mu _B$ per f.u, where the average spin and orbital moment of all Pr atoms of system-I is $1.94 \, \mu _B$ and $2.71 \, \mu _B$, respectively. The calculated magnetic moment, however, is lower than observed experimentally (section: “Isothermal magnetization”). On the other hand, the disordered unstable system-V has highest magnetic moment suggesting that quenching of moment will not be high if Co’s small moment at a specific position aligns parallel to the Pr-moment. One may note that although the disordered systems are not stable energetically at low temperature, yet such disordered structure can occur at finite temperature resulting in different moment contribution. At finite temperature, spin structure might be different for a specific system. As we have made all the calculations considering the configuration at 0 K, our prediction may not be quite exact while dealing with the finite temperature configuration, particularly at which exact positions the Co atoms couples ferromagnetically with Pr moments or even assume higher magnetic moments. Nevertheless, our calculation would be quite useful to formulate an idea that because of spin fluctuation of Pr-atoms, an unusual magnetic moment and coercivity can develop with respect to temperature. We can make prediction that at low temperature, positions of cobalt atoms in system-I provide stability to the structure.

**Table 3:** Total magnetic moment, total spin and orbital contribution and cobalt’s magnetic moment.

System	Pr-spin moment ($\mu _B$)	Pr-orbital moment ($\mu _B$)	Co-moment ($\mu _B$)	Total moment ($\mu _B$)
I	Pr1: 1.91, Pr2: 1.94	Pr1: 3.57, Pr2: 2.20	−0.14	1.692
	Pr3: 1.96, Pr4: 1.96	Pr3: 2.54, Pr4: 2.53		
II	Pr1: 1.93, Pr2: 1.95	Pr1: 2.76, Pr2: 3.01	−0.17	2.093
	Pr3: 1.92, Pr4: 1.96	Pr3: 2.86, Pr4: 2.84		
III	Pr1: 1.89, Pr2: 1.87	Pr1: 3.17, Pr2: 3.58	0.54	2.309
	Pr3: 1.88, Pr4: 1.87	Pr3: 3.27, Pr4: 3.59		
IV	Pr1: 1.95, Pr2: 1.89	Pr1: 2.39, Pr2: 3.61	−0.18	1.866
	Pr3: 1.98, Pr4: 1.97	Pr3: 2.55, Pr4: 2.54		
V	Pr1: 1.79, Pr2: 1.77	Pr1: 4.18, Pr2: 4.02	0.47	3.183
	Pr3: 1.86, Pr4: 1.86	Pr3: 3.29, Pr4: 3.29		
VI	Pr1: 1.95, Pr2: 1.94	Pr1: 2.69, Pr2: 2.55	−0.18	2.05
	Pr3: 1.95, Pr4: 1.87	Pr3: 2.42, Pr4: 3.65		

**Figure 6:**
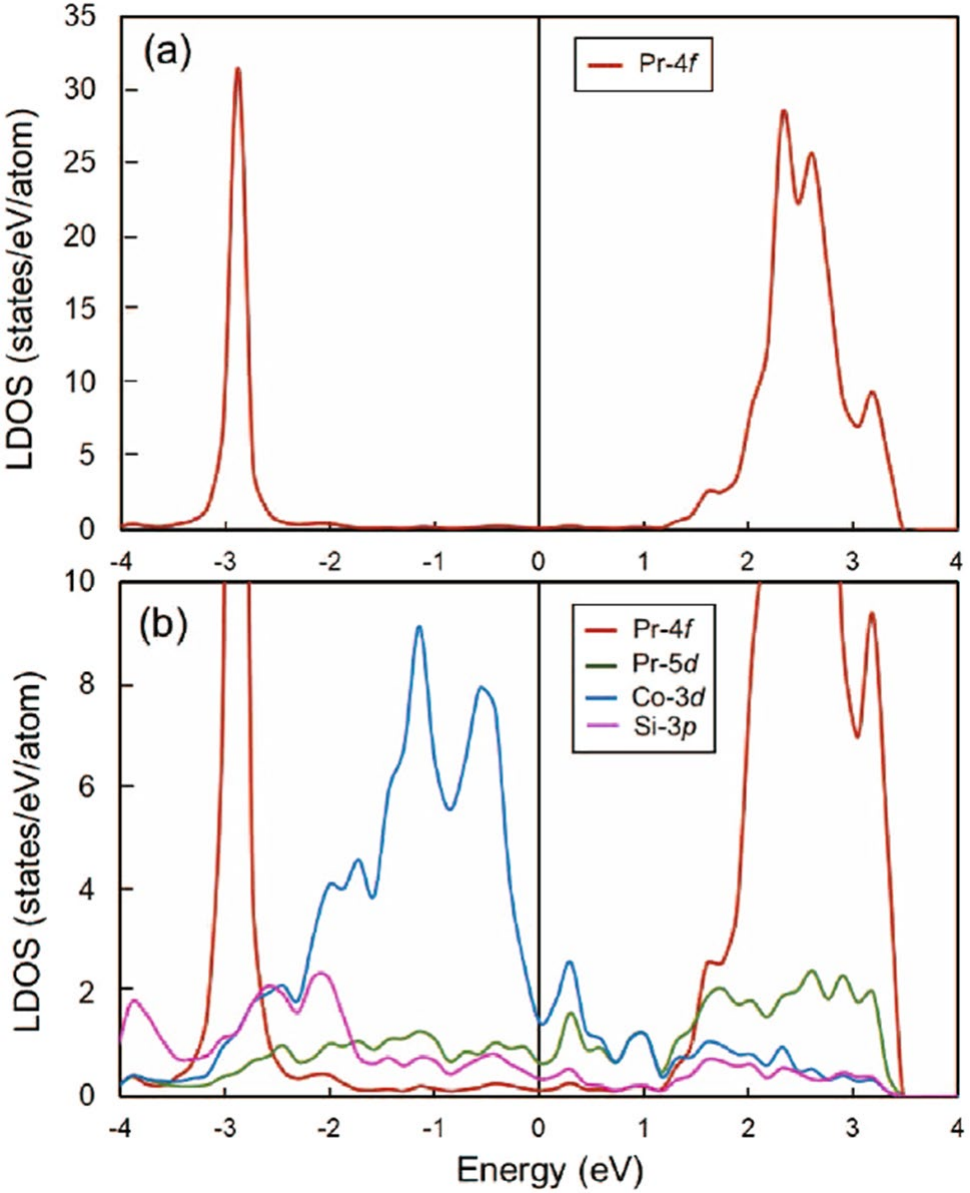
Local density of states (LDOS) of **(a)** 4*f*-states of Pr atom, and **(b)** 4*f*, 5*d* states of Pr, 3*d*-states of Co, 3*p*-states of Si atoms in system I. The Fermi level is at E = 0 eV.

According to Kramers theorem, light lanthanides—Ce, Nd, and Sm have an odd number of 4*f* electrons and crystal-field levels with even degeneracy^36^. In these, the crystal field should not in principle suppress magnetic ordering, but it should reduce the ordered moment and add magnetic complexity due to different site-symmetries. The tripositive Pr ion, with two 4*f* electrons, is a non-Kramers ion, which allows to occur singlet crystal-field states^36^. Neutron scattering experiments have revealed that both cubic and hexagonal sites in elemental Pr have a singlet as the ground state^36^. Our calculations suggest a very similar configuration in ${\text {Pr}}_2 {\text {CoSi}}_3$. The Pr in different crystallographic positions show similar 4*f* density of states (DOS) peak. As shown in Fig. 6, the 4*f* at 3 eV below the Fermi level (${\text {E}}_{\rm{F}}$), which agrees closely with the experimental peak ($\sim 2$ eV) as provided in the x-ray data booklet, show an occupied single 4*f* DOS peak. The occupied 4*f* DOS peak location in the energy axis varies depending on the on-site 4*f* electron correlation parameter used in the calculations. The decrease of the on-site 4*f* electron correlation parameter shifts the occupied 4*f* DOS peak towards higher energy. Of course, there is a substantial Pr 5*d*, Co 3*d*, and Si 3*p* hybridization at the Fermi level. We note here that if there is a substantial Pr 4*f* DOS at the Fermi level then Pr 4*f* should be in a valence fluctuating state. However, in our case, Pr 4*f* DOS at the ${\text {E}}_{\rm{F}}$ is small, which confirms Pr in the trivalent state as shown by the $\sim$2 $\mu _B$ 4*f* spin moment. One can also see a DOS peak just above the ${\text {E}}_{\rm{F}}$, which is an excited state multiplet dominated mostly by the hybridized Co 3*d* and the crystal field environment. As explained in Ref.^36^, elemental Pr, which has both hexagonal and cubic sites and in principle support $J = 4$, the lowest states of the hexagonal Pr ions (sites) are the singlet $|J_\zeta = 0>$ followed by the doublet $|J_\zeta = \pm 1>$ with an energy separation of  3–4 meV^36^. If the distortion of the point symmetry of the cubic Pr ions (sites), due to the nonideal *c*/*a* ratio, is neglected, their ground state is the $\Gamma _1$-singlet, with the $\Gamma _4$-triplet lying above the Fermi level. Borrowing the logic in our material system, the three DOS peaks (mostly originating from Pr 5*d* and Co 3*d* and hybridizing with small but non-negligible Pr 4*f* states) just above the Fermi level within 0 to 1 eV range may indicate the $\Gamma _4$-triplet whereas the hybridized DOS peak just below the ${\text {E}}_{\rm{F}}$ may represent the $\Gamma _1$-singlet. Since the coupling between Pr and Co is mostly via hybridized 5*d* and 3*d* states but not with Pr 4*f*, the concept of $J = 4$ for Pr 4*f* to provide 3 $\mu _B$ magnetic moment is no longer valid. There is also a possibility that the $\Gamma _4$ state is split into a singlet and a doublet, due to the involvement of the hexagonal and orthorhombic symmetries and also due to the hybridization between Co 3*d* and Pr 5*d*, which has rarely been seen in Pr-based systems^36^. At low temperature, only the ground state and closely lying first excited state can be populated significantly, and Pr may be considered to be a coupled singlet–doublet and singlet–triplet for both types of Pr sites. Furthermore, as explained in Ref.^36^, the difference between energy splitting in the hexagonal and cubic sites in the elemental Pr is so large, compared to the two-ion interactions, that the excitation spectrum can be divided into two parts, related respectively to the crystal-field transitions on each non-equivalent ions. The stronger crystal field interactions in Pr ion compared to the two ion interactions suggest that the effective interactions originating from Pr–Pr and Pr–Co become weak. The mapping of these interactions to temperature confirms the low temperature magnetic interactions observed experimentally. More specifically, as mentioned above, out of these two set of interactions Pr–Pr and Pr–Co, the later forms slightly stronger energetically favorable AFM alignment that effectively maps to low temperature transition. Further the weak coupling of the two components may be accounted by second-order perturbation theory, leading to an effective decoupling, with the two-ion parameters replaced by slightly different, effective values. Hence, at low temperature, Pr may be treated as a combination of a singlet–doublet and a singlet–triplet system resulting anomalous ground states in Pr-based systems due to the involvement of both hexagonal and orthorhombic point symmetries. Since there is a strong hybridization between the Co 3*d* and Pr 5*d* states and with significantly low Pr 4*f* states around the ${\text {E}}_{\rm{F}}$, and Pr (Wyckoff position : 1*a* and symmetry *6/mmm*) and Pr (Wyckoff position : 3*f* and symmetry *mmm*) representing the hexagonal and orthorhombic, respectively and Co (Wyckoff position : 2*d* and symmetry *-6m2*) representing the hexagonal sites (see Fig.  1), both singlet-doublet and singlet-triplet excited state scenarios are feasible for both Pr sites in ${\text {Pr}}_2 {\text {CoSi}}_3$. In either multiplet scenario, there is a non-negligible but small crystal-field splitting originated by Pr 4*f* around the ${\text {E}}_{\rm{F}}$. The crystal field splitting around the ${\text {E}}_{\rm{F}}$ is dominated by Co 3*d* and its hybridization to Pr 5*d*, which effectively rearranges the crystal field splitting and apparently may not allow to have $J = 4$ for Pr in ${\text {Pr}}_2 {\text {CoSi}}_3$. Instead, as mentioned above, the underlying multiplet scheme offered by the hybridized Co-3*d* and Pr-5*d* with Pr-4*f* near the ${\text {E}}_{\rm{F}}$ may correspond to an effective $J = 1$. Of course, with $J = 1$, the total 4*f* (spin and orbital) magnetic moment in Pr will not be 3 $\mu _B$ but rather 2 $\mu _B$ or even less as suggested from the DFT and experiment (section: “Isothermal magnetization”). The transformation of the $J = 4$ state of Pr to an effective $J = 1$ system introduces a scaling down of the two-ion couplings, compared to single ion allowing the magnetic moment to significantly decreased^36^ as found by DFT. The elemental Pr, which has both hexagonal and cubic sites may, therefore, support $J = 4$ and provide 4*f* magnetic moment of magnitude 3 $\mu _B$, while the coupling of Pr atom (occupied with hexagonal and orthorhombic sites) with Co atom (occupied with hexagonal sites) may transform $J = 4$ to $J = 1$ as explained in Ref.^36^. Hence the $J = 1$ model could be an appropriate low-temperature description of the Pr atoms in hexagonal and orthorhombic sites affected by the neighboring hexagonal Co sites in ${\text {Pr}}_2 {\text {CoSi}}_3$. It is therefore quite likely that the physics reflected by $J = 1$ is relevant in most of the Pr–Co based systems depending upon the crystal site symmetries and environment.

**Figure 7:**
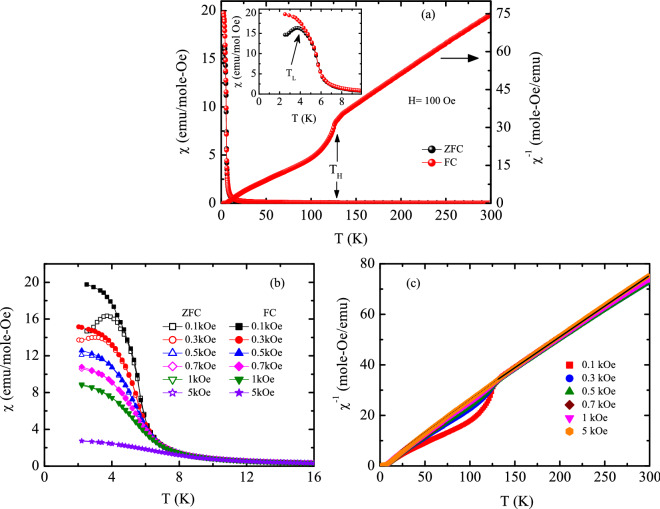
**(a)** $\chi (T)$ in ZFC and FC protocol (left panel) and $\chi ^{-1} (T)$ in FC protocol (right panel) for $H = 0.1 \, \text {kOe}$. Inset shows the expanded low-temperature region. **(b)** $\chi (T)$ and **(c)** $\chi ^{-1} (T)$ measured at various applied magnetic fields.

### DC magnetic susceptibility

The dc magnetic susceptibility ($\chi \equiv M/H$) measured in zero-field-cooled (ZFC) and field-cooled (FC) protocols for 0.1 kOe magnetic field is shown in Fig. 7(a) (left panel). The $\chi _{{\rm{ZFC}}}$ exhibits a cusp around a temperature, $T_{\rm{L}} = 4 \, \text {K}$. In contrast, $\chi _{{\rm{FC}}}$ does not exhibit any peak and monotonically increases with decreasing temperature exhibiting a saturation tendency in the lower temperature region. The $\chi _{{\rm{ZFC}}}$ and $\chi _{{\rm{FC}}}$ diverge from each other at a temperature slightly higher than $T_{\rm{L}}$. Such type of broad magnetic transition coupled with thermal irreversibility in ZFC and FC magnetization behaviour is typically reported in different magnetically frustrated systems^37,38^ as well as in superparamagnetic systems. It should be mentioned here that low temperature magnetic ground state of many other ${\text {R}}_2 {\text {TX}}_3$ type of materials exhibit coexistence of spin-glass/cluster-glass behaviour and spatially limited magnetically ordered phase^1,18^. Since in these type of compounds R ions are reported to order in the low temperature region^7,18,39–41^, this ordering at 4 K is apparently linked to 4*f* moment associated with Pr-ions present in the ${\text {Pr}}_2 {\text {Co}}_{0.86} {\text {Si}}_{2.88}$ system, although one can not rule out the contribution of Co moments. The DFT calculations (discussed earlier in section: “Theoretical investigation”) indeed suggest that the ordering below 4 K is associated with AFM coupling between sublattices containing Pr moments and Co moments. DFT calculations also suggest that the coupling between Pr 4*f* electrons is very weak in nature and hence can not stabilize any long-range magnetic ordering among itself. Magnetic susceptibility measured under different applied magnetic fields are plotted in Fig. 7(b). As seen from the figure, a magnetic field, as small as 1 kOe, is sufficient to remove the irreversibility of magnetic susceptibility by showing a FM type characteristics for both $\chi _{{\rm{ZFC}}}(T)$ and $\chi _{\rm{FC}}(T)$. Such behaviour indicate the fragile nature of magnetic ground state in ${\text {Pr}}_2 {\text {Co}}_{0.86} {\text {Si}}_{2.88}$ and quite similar to that observed in many magnetically frustrated systems including glassy and superparamagnetic systems^12,18,42,43^. A careful examination of the magnetization data reveals a signature of an additional, but very weak magnetic phase transition around 120 K ($T_{\rm{H}}$) as could be clearly visualized from the inverse susceptibility behaviour plotted in Fig. 7(c). This feature too gets suppressed at higher magnetic fields and vanishes above $H \sim 1 \, \text {kOe}$. Such a weak signature of spin ordering can be originated due to small impurity present in the system or it may result from the ordering of the 3*d* moment or formation of magnetic cluster inherent in the system. However, if it is an impurity effect, we are left with a rather unrealistic situation where the primary and secondary phase appear to co-exist in a microscopically interpenetrating manner as indicated by the isothermal magnetization (section: “Isothermal magnetization”) and $\mu$SR data (section: “$\mu$SR experiment”). The XRD and EDX result combined with SEM-BSE image analysis also confirm the essentially bulk single phase nature of the synthesized material. Thus, a more probable hypothesis would be to associate the high temperature magnetic phase with the spin of the *d*-element (Co) present in ${\text {Pr}}_2 {\text {Co}}_{0.86} {\text {Si}}_{2.88}$. To examine, whether any indication of the presence of Co-moment is reflected in the effective moment value of the system, the inverse susceptibility data in the high temperature region have been fitted using Curie-Weiss law. The fitting yields an effective moment $\mu _{eff}= 5.78~\mu _B/\text {f.u.}$ If we consider that in our system, Pr-ion is the only one that contributes to magnetization, then the effective moment per Pr-ion would take the value $\mu _{eff}= 4.08~\mu _B$. This value of $\mu _{eff}$ is quite higher than $3.58 \, \mu _{B}$, expected for free ${\text {Pr}}^{3+}$ ion. Higher estimated value of $\mu _{eff}$ also suggests the contribution of *d* electrons in the magnetization having itinerant character^44^. The Weiss temperature comes out to be -14.5 K *i.e.* $\theta _{\rm{P}}\sim \text {-}3 {\text {T}}_{\rm{L}}$ indicating presence of a significant magnetic frustration in the system^45^ that, however, gets weakened with increasing magnetic field. The negative value of the Weiss temperature indicates the presence of dominating antiferromagnetic interaction in the system. The dc magnetic susceptibility measurements along with DFT calculations clearly indicate that the system undergoes two magnetic transitions; one below 120 K associated with FM cluster formation of itinerant Co moments and another below 4 K caused by AFM coupling between localized Pr 4*f* electron sublattice with itinerant Co 3*d* electron sublattice. The observed negative $\theta _{\rm{P}}$ is a possible manifestation of the AFM coupling between Pr and Co spins.

**Figure 8:**
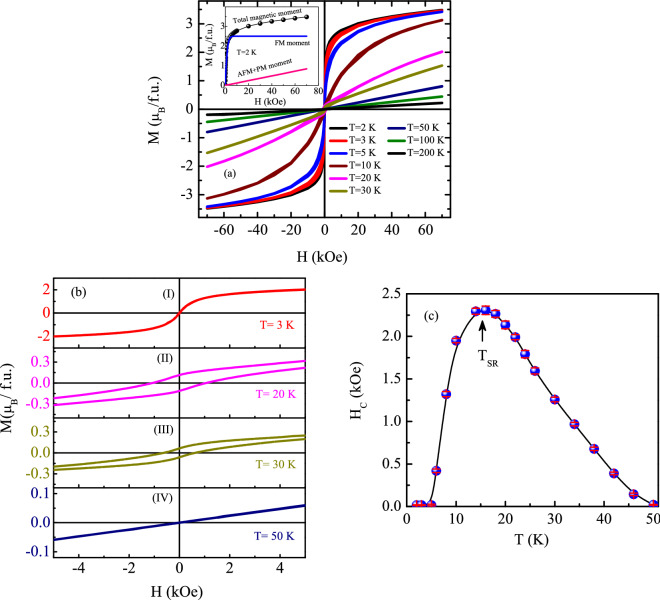
**(a)** Magnetic field dependence of magnetization at different temperatures. Inset shows the isothermal magnetization curve at $T = 2 \, \text {K}$ along with the estimated FM and AFM/PM contributions. **(b)** Low field region of isothermal magnetization at some selected temperatures, $T = 3 \, \text {K}$ (I), 20 K (II), 30 K (III), and 50 K (IV). **(c)** Temperature dependence of coercive field. Solid line is guide to the eye.

### Isothermal magnetization

The field evolution of the magnetic ground state has been studied through magnetic field dependent isothermal magnetization measurements at different temperatures. The signature of field induced ferromagnetic type ordering observed in the magnetic susceptibility measurement at low temperature, is reflected more prominently in the isothermal magnetization measurement as shown in Fig. 8(a). For a system with antiferromagnetic interaction over the measured field range, a linear *M*(*H*) behaviour is generally expected. However, an overall ‘*S*’-shape nature^46–48^ is seen in the isothermal magnetization of ${\text {Pr}}_2 {\text {Co}}_{0.86} {\text {Si}}_{2.88}$, where linear field dependency is observed for low *H* and a saturation tendency is observed for higher *H*. At 2 K (lowest measured temperature), the *M*(*H*) curve exhibits a sharp rise in the low field region and tends to saturate for $H \ge 2$ kOe. The magnetization reaches a value of $\sim$ 3.48(2) $\mu _B$/f.u. when subjected to $H = 70 \, \text {kOe}$. If Pr would be the only moment contributing element in our system, saturation moment should reach to a value of $6.4 \, \mu _B/\text {f.u.}$ as $gJ = 3.2 \, \mu _B/\text {Pr-ion}$. As the DFT calculation suggests, it is quite likely that either the antiferromagnetic coupling of Pr and Co atoms at low temperature (below $T_{\rm{L}}$) and/or orbital quenching of Pr moment and/or CEF effect are/is responsible for the reduction of saturation magnetic moment.

The low temperature magnetic isotherms could be described well by considering a tangent-hyperbolic of Brillouin function (representing the ferromagnetic contribution) together with a linear term (describing antiferromagnetic and/or paramagnetic behavior) in the applied field range, $0 \le ~H~\le 70\, \text {kOe}$ (Inset: Fig. 8(a)). No magnetic hysteresis is found in the isothermal magnetization data measured below $T_{\rm{L}}$. The non-linear nature of *M*(*H*) isotherms persist up to very high temperature ($T \sim 50\, \text {K}$) that is at least one order higher in magnitude than $T_{\rm{L}}$ ($\sim$ 4 K) of this compound. Though magnetic susceptibility data reveals another weak magnetic transition in ${\text {Pr}}_2 {\text {Co}}_{0.86} {\text {Si}}_{2.88}$ below 120 K, the *M*(*H*) behaviour is found to be almost linear in the temperature range $50\, \text {K} \le T\le 120\, \text {K}$. The absence of any profound signature of spin ordering in the isothermal magnetization measurements in that temperature region might be due to a very weak nature of ordered spins associated with small isolated itinerant Co moments clusters rather than any long-range magnetic spin arrangement. As the temperature is lowered below 50 K, the magnetic interaction among the clusters become significant to yield a nonlinear *M*(*H*) behaviour. Figure 8(b) depicts magnetic hysteresis curves of ${\text {Pr}}_2 {\text {Co}}_{0.86} {\text {Si}}_{2.88}$ at few selected temperatures in the expanded scale close to the low field region. A very interesting observation is that nonlinear *M*(*H*) behaviour is also associated with finite coercivity ($H_c$) for $T < 50 \, \text {K}$, further signifying the considerable growth of FM clusters. The $H_c$ gradually increases with further decrease in temperature down to the temperature, $T_{\rm{SR}}\sim 16 \, \text {K}$, below which it drops to zero at a rather faster pace (Fig. 8(c)). This observation is very unusual and certainly puzzling too. The $H_c$ behaviour for $T \ge T_{\rm{SR}}$ is quite general to that observed in most ferromagnetic or glassy systems^49–51^. However, sudden drop in $H_c$ value below $T_{\rm{SR}}$ strongly indicates the appearance of another magnetic interaction that competes with that belonging to the magnetic phase of higher temperature. This unusual behaviour of $H_c (T)$ also rules out any impurity effect for the magnetic ordering seen at high temperature. It is quite possible that the high temperature magnetic phase associated with itinerant Co moment (3*d*) compete with the development of localized Pr (4*f*) moments before settling for an antiferromagnetic ordering below $\sim 4 \, \text {K}$. The growth of localized Pr moments around $T_{\rm{SR}}$ strongly suppress the magnetic coercivity associated with the FM clusters of Co moments which are itinerant in nature resulting an unusual behaviour of coercivity with respect to temperature. Such a behaviour of $H_c$ is quite rare for polycrystalline intermetallics, but a nearly similar response can be seen in some systems including thin film, nanoparticle and R-cobalt magnets due to different reasons^52–58^. For example, the anomalous temperature dependence of Co-antidot array has been explained in terms of magnetocrystalline anisotropy and pinning of the pore^58^, whereas, in hardened Pr–Co–Cu–Ti magnets, domain wall pinning is caused by cellular microstructure^57^. The observation of coercivity in our system indicates that the high temperature phase is associated with a domain structure, and the domain wall motions are pinned to the system. It means that with the application of field, the domain walls of the systems moves however it is not possible to get the domain walls back to the initial position even after withdrawing the magnetic field. The pinning mechanism gets stronger with decreasing temperature below $\sim$ 50 K, resulting the enhancement of coercive field, typically observed in both ferromagnetic as well as glassy systems^59–62^. The rather unusual feature of sudden drop in coercivity below $T_{\rm{SR}}$, could be due to gradual diminution of the domain wall pinning strength, which vanishes completely below $T_{\rm{L}}$. Since the short-range correlation associated with localized moments starts to develop below $T_{\rm{SR}}$ (section: “Heat capacity”) and exhibit an ordering below $T_{\rm{L}}$, it is quite likely that the growth of moment that induces the low temperature magnetic ordering, is responsible for the destruction of pinning mechanism of the block-walls of high temperature magnetic phase.

It may be pointed out here that the DFT calculation suggests a singlet ground state of ${\text {Pr}}^{3+}$ ions, which carries no magnetic moment. To consider the magnetic ordering in such systems, one needs to go beyond the single-ion crystalline electric field (CEF) level scheme, and instead consider the two-ion exchange interactions. The magnetic moment in these systems can only be induced by the CEF transition to a low-lying excited state. In such case, an effective ground state is formed by the coupling of singlet ground state with the first excited state, which may be a doublet or a triplet. A similar exciton-like CEF transition had earlier been reported to induce magnetic transition in ${\text {PrNi}}_2 {\text {B}}_2 \text {C}$, that also have a singlet ground state^63^. Our DFT calculation (section: “Theoretical investigation”) has also suggested a similar singlet–doublet and a singlet–triplet scenario in ${\text {Pr}}_2 {\text {CoSi}}_3$ system. From the temperature dependent magnetic hysteresis behaviour, it appears that the first excited state in ${\text {Pr}}_2 {\text {Co}}_{0.86} {\text {Si}}_{2.88}$ lies at around $T_{\rm{SR}}\sim \, 16 \, \text {K}$ ($\sim$1.5 meV) above the ground state. As the system temperature goes below $T_{\rm{SR}}$, the formation of the effective magnetic ground state starts to introduce Pr magnetic moment in the interwoven space of Co-spins. The occurrence of Pr-spins in the intermediate space disturbs the domain structure of Co-spins, gradually changing the Co-spin arrangements towards a softer ferromagnetic-type. Below 4 K, the ferromagnetic Co-spins gets antiferromagnetically coupled with the Pr-spins having a combined singlet-triplet or singlet/doublet ground state.

**Figure 9:**
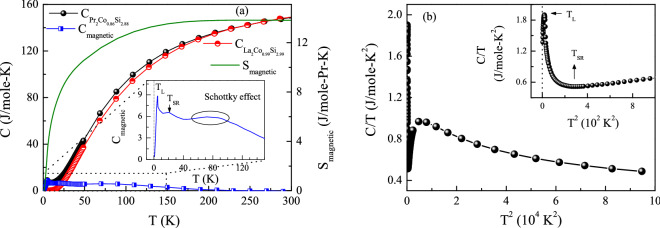
**(a)** Zero-field heat capacity of ${\text {Pr}}_2 {\text {Co}}_{0.86} {\text {Si}}_{2.88}$, its non-magnetic counterpart ${\text {La}}_2 {\text {Co}}_{0.99} {\text {Si}}_{2.99}$, along with the magnetic component in heat capacity of the former (left panel) and magnetic entropy (right panel) as a function of temperature. Inset shows the expanded view of magnetic contribution for a limited range of temperature. **(b)** Heat capacity data in the form of *C*/*T* as a function of $T^2$. Inset shows the expanded view in the low temperature region.

### Heat capacity

The heat capacity measurement, $C_{\rm{p}}(T)$, is a very useful technique to investigate the magnetic ordering phenomenon in a material. It not only gives indication to the magnetic ordering temperature, but can also be used to estimate the magnetic entropy, which in turn can provide information on CEF effect as well as a clue for the fraction of magnetic ions taking part in magnetic ordering process. The zero-field heat capacity measurement has been carried out over the temperature range of 2–300 K in order to understand the signatures of magnetic interactions at different temperatures revealed through magnetization measurements. The occurrence of a $\lambda$-like peak around 4.5 K confirms the magnetic transition observed in the magnetic measurement described earlier. However, the height of that peak appears to be substantially reduced in comparison to those reported earlier in many other Pr-based magnetic systems^64–66^. The specific heat value attains a saturation value of $\sim$147 J/mole-K near room temperature (Fig. 9(a)) that is very close to the classical Dulong-Petit limit value, $C = 3nR$, where *n* = number of atoms per formula unit (here $n~=~2+0.95+2.96~=~5.91$ obtained from Full-Rietveld analysis) and *R* is the universal gas constant ($8.314 \, {\text {J mole}}^{-1} {\text {K}}^{-1}$). Although the development of Pr-spin below $T_{\rm{SR}} \sim 14\text {-}16 \, \text {K}$ could not be directly discernible from the $C_{\rm{p}}(T)$ behavior, the phenomenon gets manifested clearly when the data is plotted in $C_{\rm{p}}(T)/T$ vs. $T^2$ representation (Fig. 9(b)).

The magnetic contribution of ${\text {Pr}}_2 {\text {Co}}_{0.86} {\text {Si}}_{2.88}$ has been estimated by subtracting the $C_{\rm{p}}(T)$ data of non-magnetic analogue ${\text {La}}_2 {\text {Co}}_{0.99} {\text {Si}}_{2.99}$, after appropriately considering the effect of the difference of formula weights of these two compounds. The molar mass-corrected lattice contribution can be obtained by scaling^67^ the measured temperature (*T*) in ${\text {La}}_2 {\text {Co}}_{0.99} {\text {Si}}_{2.99}$ as

3 $$\begin{aligned} T^*\, = \, & {} \frac{T}{(M_{Pr_2Co_{0.95}Si_{2.96}}/M_{La_2Co_{0.99}Si_{2.99}})^{1/2}} \end{aligned}$$

It can be seen from Fig. 9(a) that, the $C_{\rm{p}}(T)$ data of ${\text {Pr}}_2 {\text {Co}}_{0.86} {\text {Si}}_{2.88}$ coincides with that of ${\text {La}}_2 {\text {Co}}_{0.99} {\text {Si}}_{2.99}$ in the high temperature region, but starts to deviate from each other slightly below $\sim$165 K. This large discrepancy may originate either due to the presence of magnetic interaction in ${\text {Pr}}_2 {\text {Co}}_{0.86} {\text {Si}}_{2.88}$ up to such high temperature, or as a result of CEF effect of Pr-ions, or due to the presence of both. The calculated magnetic part ($C_{\rm{mag}}(T)$) shows a small peak around $\sim 4.5$ K due to the low temperature magnetic transition in addition to a broad hump around the high temperature region $\sim$ 80 K which is slightly below $T_{\rm{H}}$ (Inset: Fig. 9(a)). A similar nature of hump in $C_{\rm{mag}}(T)$, is generally attributed to the Schottky anomaly originating due to the CEF splitting of ground state energy levels^68–70^. Additionally, a small kink is observed around $T \sim 20 \, \text {K}$ in $C_{\rm{mag}}(T)$ (Inset: Fig. 9(a)), which is very close to $T_{\rm{SR}}$ estimated from the magnetization measurement. It may be pointed out here again that the variation of $H_c$ with *T* exhibits a peak-like structure (Fig. 8(c)) around the same temperature. The magnetic contribution to entropy of the system has been estimated as

4 $$\begin{aligned} S_{mag}(T)\, = \, & {} \int _{0}^{T}\frac{C_{mag}(T)}{T} dT \end{aligned}$$

Although the minimum magnetic entropy ($S_{\rm{mag}}$) required for the occurrence of long-range ordering in a system is *Rln*2 (for doublet ground state), in case of ${\text {Pr}}_2 {\text {Co}}_{0.86} {\text {Si}}_{2.88}$ it is about 48(1)% of that around $T_{\rm{L}}$. Such low value of $S_{\rm{mag}}$ is not surprising as in this case, $T_{\rm{L}}$ is not solely associated with 4*f* moment, rather it defines an AFM coupling between itinerant 3*d* and localized Pr 4*f* moment. Additionally, the magnetic entropy near room temperature attains only 76(1)% of its expected saturation value $Rln(2J+1)$ with $J = 4$ for ${\text {Pr}}^{3+}$ ion. Failure of full entropy release even at room temperature might be due to the presence of the earlier mentioned short-range magnetic interaction up to high temperature or CEF or presence of both of these two in the system^28,71^.

The heat capacity data of ${\text {Pr}}_2 {\text {Co}}_{0.86} {\text {Si}}_{2.88}$ have also been analyzed using the relation

5 $$\begin{aligned} C(T)\, = \, & {} \gamma T+C_{\rm{D}}(T) \end{aligned}$$

where $\gamma T$ is the electronic contribution and the second term represents the Debye lattice heat capacity.

**Figure 10:**
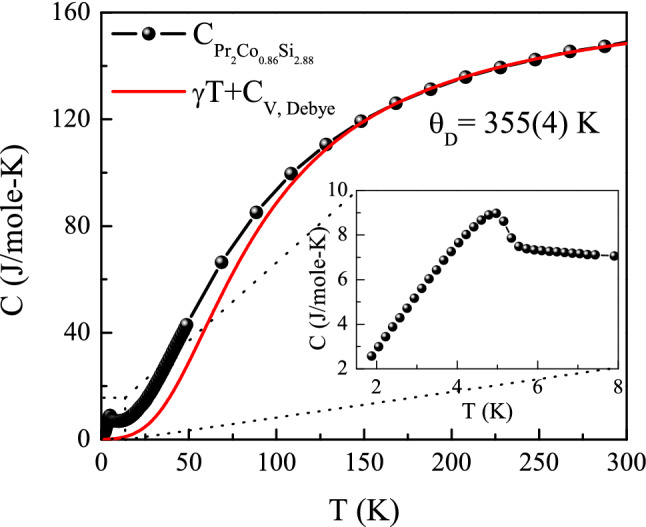
Heat capacity of ${\text {Pr}}_2 {\text {Co}}_{0.86} {\text {Si}}_{2.88}$ fitted in the temperature range 165-300 K, using Eq. (5) and extrapolated down to 0 K. Inset presents the experimental *C*(*T*) data in the region of magnetic ordering.

According to Debye model, $C_{\rm{D}} (T)$ could be written as

6 $$\begin{aligned} C_{\rm{D}}(T)\, = \, & {} 9nR{\left( \frac{T}{\theta _{\rm{D}}}\right) }^3\int _{0}^{\theta _{\rm{D}}/T}\frac{x^4e^x}{(e^x-1)^2} dx \end{aligned}$$

where *n* is the number of atoms per formula unit and $x=\theta _{\rm{D}}/T$ and $\theta _{\rm{D}}$ is the Debye temperature. A good fit can be achieved in the temperature range 165–300 K with $\gamma =0.029 \, {\text {J mole}}^{-1} \, {\text {K}}^{-2}$ and Debye temperature $\theta _{\rm{D}}= 355(4)$ K, which is a little higher than that obtained from low temperature XRD analysis. In this analysis too, one may notice additional contribution of heat capacity up to $\sim$120 K, over and above the theoretically estimated lattice and electronic contribution (Fig. 10). Therefore, it is very likely that the enhanced contribution to heat capacity upto $\sim$120 K could be a result of a short-range type magnetic interaction associated with Co-ions. Though the presence of magnetic moment carrying transition metal ions in rare-earth intermetallic systems have also been reported in earlier literatures^21,44,72–77^, it is not very commonly observed in ${\text {R}}_2 {\text {TX}}_3$ series of compounds except in systems like ${\text {Ce}}_2 {\text {CoSi}}_3$^21^, *etc*. This will be discussed further in detail in “section: $\mu$SR experiment”.

**Figure 11:**
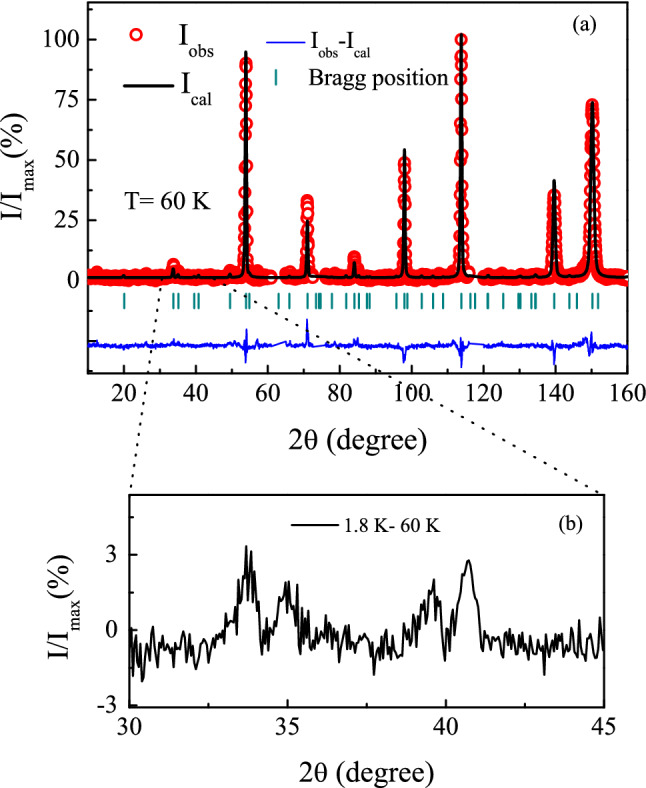
**(a)** Powder neutron diffraction data at 60 K fitted using full-Rietveld analysis with $\lambda = 2.4395~{\AA }$. Diffraction peaks due to aluminium sample holder are excluded. **(b)** Magnetic peaks in a selected region as found in the spectra measured at 1.8 K.

### Neutron diffraction

Neutron diffraction (ND) measurements ($\lambda = 2.4395~{\AA }$) have been carried out at different temperatures to investigate the magnetic structure of the system. The data have been taken at four different temperatures: $T = 1.8, 4.3, 20$ and 60 K. The diffraction pattern for $T = 60 \, \text {K}$, can be fitted well through the standard full-Rietveld analysis by considering the crystal structure only (Fig. 11(a)). The pattern does not exhibit any significant change even when we reduce the temperature to 1.8 K. Nevertheless, on close investigation, particularly when 60 K or 20 K data is subtracted from the 1.8 K or even the 4.3 K spectra, one may indeed notice very weak enhancement of a few peaks as well as development of some additional peaks which are not allowed in crystal Bragg positions (Fig. 11(b)). The weak nature of the magnetic Bragg peaks could be understood if we consider the corresponding magnetic ordering to be associated with the antiferromagnetic interactions among the itinerant Co 3*d*-spins of quite low moments ($\sim 0.14~\mu _B$) and local (4*f*) moments of Pr with a singlet-doublet or singlet-triplet ground state exhibiting spin-fluctuation behaviour, as suggested in our theoretical calculations (section: “Theoretical investigation”). The growth of such magnetic Bragg peaks, however weak, suggests the development of AFM in addition with possible FM components below $\sim$ 5 K. It may be noted here that quite a few other intermetallic compounds of similar structure are known to exhibit multiple magnetic phases^18,78,79^. The magnetic peaks are very weak, may be due to Pr–Co coupling which restricts magnetic moments to develop to its full potential below the ordering temperature $\sim$5 K. As a result, the exact magnetic structure(s) of ${\text {Pr}}_2 {\text {Co}}_{0.86} {\text {Si}}_{2.88}$ could not be determined from ND experiment.

### $\mu$SR experiment

The magnetic susceptibility measurement had earlier suggested the formation of an ordered/glassy phase below $T_{\rm{H}} \sim 120 \, \text {K}$, while the heat capacity measurement also showed a signature of magnetic entropy below $\sim$160 K. From the temperature dependent isothermal magnetization measurement we have suggested the high temperature magnetic phase to be of intrinsic origin, i.e.﻿ a contribution from Co-moment gradually developing a short-range character below 50 K. Additionally, susceptibility, heat capacity measurements and DFT calculations suggest a Pr-Co magnetic ordering below 4 K but with weak ordered moment as revealed through neutron diffraction measurement. To understand the nature of magnetism in a clearer way, we have carried out $\mu$SR measurements over a wide temperature range $2 \, \text {K} \le T\le 190 \, \text {K}$. Because of large gyromagnetic ratio ($\gamma _\mu$) and the full polarization of the muons, $\mu$SR is sufficiently sensitive to track static internal fields as low as a fraction of a Gauss (G). During the experiment, muon beams (with a known initial moment direction) are stopped in the powdered sample of interest, that was mounted on a silver plate. During muon’s lifetime ($\sim \, 2.2 \, \mu \text {s}$) in the sample, the muon spins precess and loose their initial polarisation because of the presence of static and/or dynamic internal fields. In a $\mu$SR experiment, the required magnetic information are thus obtained by measuring the time dependence of the relaxation of muon polarization. $\mu$SR relies on the asymmetry of the positron emission, being emitted preferentially in the direction of muon spin just before its decay. Thus to measure the time evolution of the muon spin polarisation within the system, emitted positrons were collected in the forward (F) and backward (B) detector arrays relative to the initial muon spin direction. By calculating the asymmetry parameter $\text {A}(t)= [\text {F}(t)\text {-} \alpha \text {B}(t)]/ [\text {F}(t)+ \alpha \text {B}(t)]$, where $\alpha$ is a calibration constant relating to detector efficiency that can be estimated from the 20 G transverse field (TF) data at high temperature, one can remove the lifetime decay^80–82^. If the system of interest is crystallographically perfect and have long-range magnetic order, all muons stopping at the same crystallographic sites experience the same local field, resulting a spontaneous oscillatory time dependence of polarisation in zero external field and the envelope of the signal provides information on the field distribution itself^83^. The frequency of this oscillation is a direct measurement of internal field. However, if the system is structurally and/or magnetically disordered, rather than seeing a single unique field, the implanted muons are subjected to field that are distributed in magnitude. This washes out the simple precession and leads to form with a single minimum^80,84,85^. The position of this minimum is related to the average field at the muon stopping sites.

**Figure 12:**
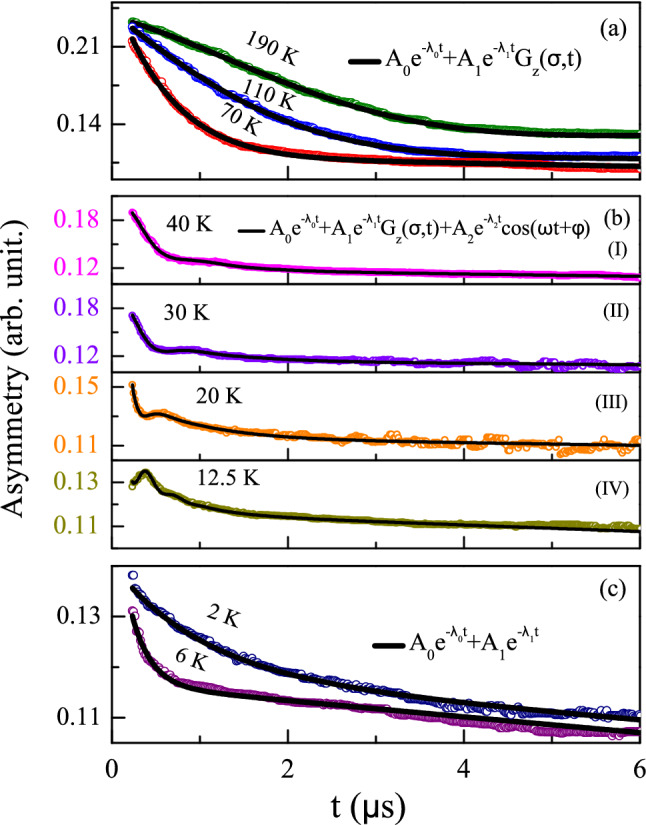
Zero-field $\mu$SR spectra **(a)** at temperatures that can be fitted using Eq. (7), **(b)** at temperatures that can be well described using Eq. (9) and **(c)** at temperatures where the fitting equation (Eq. (7)) must have $\sigma =0$.

**Figure 13:**
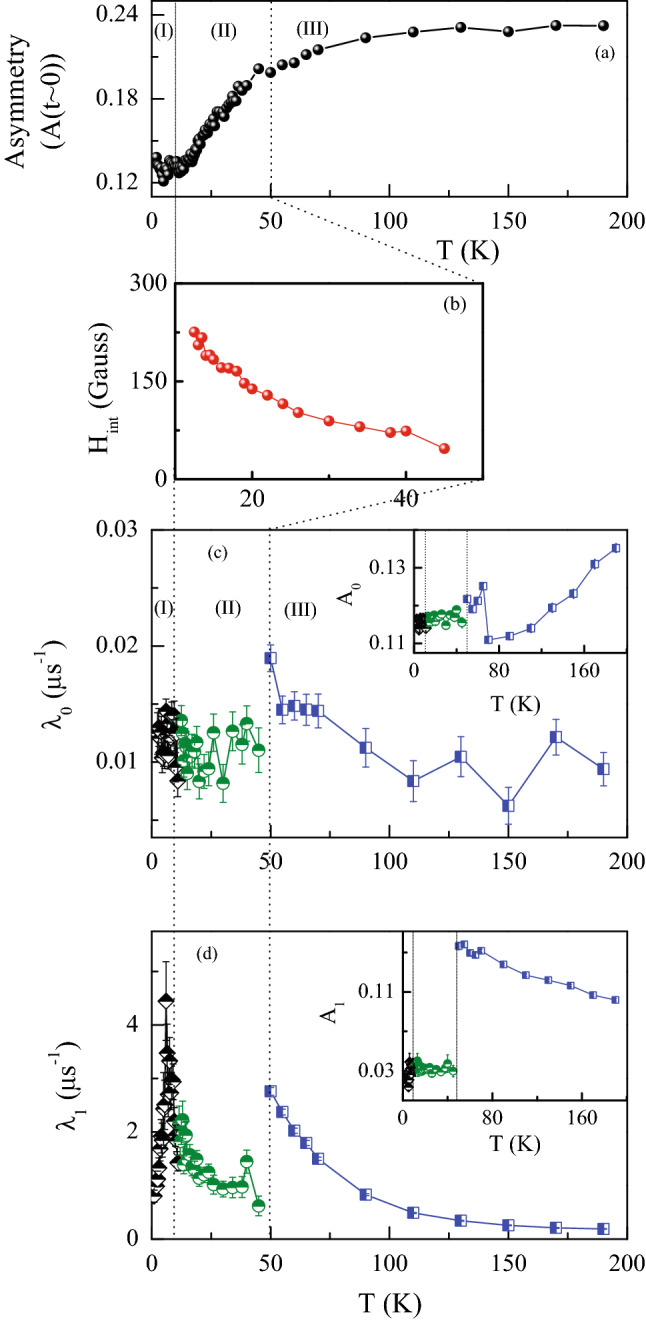
**(a)** Variation of total asymmetry at $t\sim 0 \, \mu \text {s}$ from the raw spectra. **(b)** Variation of internal field (${\text {H}}_{\rm{int}}$) with temperature. **(c)** Temperature variation of $\lambda _0$ and corresponding asymmetry ${\text {A}}_0$ is shown as inset. Region (I), (II) and (III) are described using Eq. (7) with $\sigma =0$, Eqs. (9) and  (7) respectively. **(d)** Temperature dependence of $\lambda _1$ and the inset presents the variation of ${\text {A}}_1$.

The time dependence of asymmetry measured at zero-field for different temperatures is shown in Fig. 12. The data are grouped (a,b,c) in three broad temperature regions. At high temperatures ($190 \, \text {K} \, \ge T\ge \, 50 \, \text {K}$), the nature of asymmetry is shown in Fig. 12(a). Below 50 K, an oscillatory signal develops (Fig. 12(b)) for the system. When the temperature is decreased further below 12 K, the nature of the asymmetry again changes in such a way that the oscillation is not visible in the temperature range $12 \, \text {K} \ge T\ge 2 \, \text {K}$ (Fig. 12(c)). Interestingly, the overall behaviour of temperature dependent initial asymmetry ($t\sim$ 0 $\mu$s) of the raw $\mu$SR data (Fig. 13(a)) tend to exhibit a gradual loss in magnitude below $T_{\rm{H}}\sim 120 \, \text {K}$, suggesting a slow growth of ordered/glassy moment in the system. From the magnetic measurements presented earlier, we have suggested a gradual formation and development of magnetic clusters consisting of Co-*d*-electrons below $T_{\rm{H}}$. The signature of cluster formation or short-range interaction have also been observed in the heat capacity data as discussed in “section: Heat capacity”. It may also be noted that while the isothermal magnetization measurements start to exhibit hysteresis behaviour below 50 K (see Fig. 8), oscillatory component also starts to develop in the $\mu$SR spectra below the same temperature.

Depending on the nature of the time dependent asymmetry, the $\mu$SR data in the three temperature regions have been analysed using different equations. These equations are chosen in their simplest forms in order to describe the behaviour of the signal together with the underlying physics, with the minimum number of parameters. The asymmetry in the temperature range $190 \, \text {K} \ge T\ge 50\, \text {K}$ (Fig. 12(a)) can be analysed using a two components function, given below

7 $$\begin{aligned} A(t)\, = \, & {} A_{0}e^{-\lambda _{0}t}+A_{1}e^{-\lambda _{1}t}G_{z}^{KT}(\sigma ,t) \end{aligned}$$

Here the first term presents the slow relaxation component that also includes a small background contribution from the silver plate. The second term represents the fast depolarizing component, associated with muons sensing electronic spin fluctuations while coupling to nuclear moments in the material. Here $A_0/A_1$ is the initial asymmetry and $\lambda _0/\lambda _1$ is the depolarization rate. $G_z^{KT}(\sigma ,t)$ represents the Kubo-Toyabe (KT) function with nuclear depolarization rate $\sigma$ that describes the effect of nuclear dipole fields distributed at the muon site. The functional form of $G_z^{KT}(\sigma ,t)$ is expressed as^86–88^

8 $$\begin{aligned} G_z^{KT}(\sigma ,t)\, = \, & {} \frac{1}{3}+\frac{2}{3}(1-\sigma ^2t^2)exp \left( -\frac{\sigma ^2t^2}{2}\right) \end{aligned}$$

$\sigma /\gamma _\mu = \Delta$ is the local Gaussian field distribution width and $\gamma _\mu$ is the gyromagnetic ratio of the muon. The nuclear depolarization rate $\sigma$ in this temperature range, remains constant at $\sim 0.335 \, \upmu {\text {s}}^{-1}$.

Below 50 K, the depolarizing component comes with a muon-spin precession that can be followed down to $\sim$ 12 K (which is slightly lower than $T_{\rm{SR}} \sim 16$ K estimated from the *C*(*T*) measurements). The time variant asymmetry parameter in the temperature range $50\, \text {K} \ge T\ge 12\, \text {K}$ can be well fitted using

9 $$\begin{aligned} A(t)\, = \, & {} A_{0}e^{-\lambda _{0}t}+ A_{1}e^{-\lambda _{1}t}G_{z}^{KT}(\sigma ,t) \nonumber \\&+ A_{2}e^{-\lambda _{2}t}cos(wt+\phi ) \end{aligned}$$

where the third term describes the spin precession of muons with frequency *w* and phase $\phi$^80,88–90^. One can estimate the internal field ${\text {H}}_{\rm{int}}$ at the muon site from the parameter *w*, using ${\text {H}}_{\rm{int}}=w/\gamma _\mu$, where $\gamma _\mu$ is the gyromagnetic ratio of muon ($\gamma _\mu = 13.55 \, \text {MHz/kOe}$). Fig. 13(b) shows that the internal field ${\text {H}}_{\rm{int}}$ increases with decreasing temperature. As the temperature is reduced further below 12 K, the oscillatory behaviour is no more apparent (Fig. 12(c)). It is quite likely that below $T_{\rm{SR}}$, due to the development of short-range interaction among Pr-Co magnetic ions (leading to a magnetic ordering at $\sim$ 4 K), the effective local internal field grows so large that the frequency of oscillation falls outside the finite time window accessible by $\mu$SR spectrometer^82,89^ at ISIS facility. As the internal field due to electronic moment dominates over weak nuclear-dipole field at the muon site near transition $T_{\rm{L}}$, $\sigma =0$ effectively^90^, thus in this temperature range ($12 \, \text {K} \ge T\ge 2 \, \text {K}$) the data can be fitted using Eq. (7), with $G_z^{KT}(\sigma ,t)=1$ and the oscillatory component is removed as there is no clear sign of oscillations in this temperature range.

The temperature dependence of the relaxation rate, $\lambda _0$, and the asymmetry, ${\text {A}}_0$ (inset), are shown in Fig. 13(c) for the temperature range $2 \, \text {K} \le T\le 190 \, \text {K}$, with similar data for $\lambda _1$ and ${\text {A}}_1$ presented in Fig. 13(d). There is a sudden drop in the value of ${\text {A}}_1$ around 50 K, as can be seen from Fig. 13(d). This is in agreement with the coercive field seen below 50 K, suggesting a development of magnetic cluster with finite FM component. Additionally, a close look in the data reveals that there is another sudden drop in ${\text {A}}_1$ around $\sim$ 5 K i.e. around $T_{\rm{L}}$ although it does not follow the $1/3^{rd}$ rule which is true for long-range ordering^78,80,85,91^. The temperature dependence of depolarization rate, $\lambda _1$, also reflects the signature of both the magnetic interactions. We have also carried out longitudinal field (LF) dependent measurements at 1.7 K, 20 K and 65 K and 190 K (data not shown here, but given in the supplementary materials). Small LF (50 G) were applied to confirm the origin of the spin relaxation measured. At 65 K and 190 K the relaxation was partially quenched by the applied field as nuclear fields were decoupled; however, even at these temperatures, quite above the magnetic transitions, significant depolarisation due to fluctuating electronic moments was still seen. At lower temperatures the 50 G LF had minimal effect as electronic moments dominate in this regime. High field decoupling measurements (4000 G) were also carried out at the three lower temperatures; in each case full asymmetry was recovered at the highest fields, although significant spin relaxation due to the presence of electronic moments remained.

## Conclusions

In conclusion, we report the successful synthesis and physical properties of a novel material ${\text {Pr}}_2 {\text {Co}}_{0.86} {\text {Si}}_{2.88}$ exhibiting an anomalous temperature dependence of magnetic coercivity associated with complex interplay of local and itinerant magnetism, present in the system. The magnetization, heat capacity as well as $\mu$SR measurements indicate the development of a magnetic phase below $\sim$ 120 K with an additional low temperature ordering at 4 K, although no signature of any long-range magnetic ordering is evidenced from zero-field neutron diffraction studies. On the basis of experimental evidences along with theoretical investigation we have shown that the high temperature magnetic transition is associated with itinerant Co 3*d* moments and short-range in nature, while antiferromagnetic coupling between localized Pr 4*f* moments with Co 3*d* moments result in another short-range magnetic ordering below 4 K. The presence of magnetic moments on Pr as well as Co atoms and their mutual interaction leads to a very unusual behaviour of temperature dependence of magnetic coercive field which, with decreasing temperature, gradually develops below 50 K, but starts to reduce its strength below 16 K before vanishing completely below 5 K. By performing a number of different experiments, we have argued that the magnetic coercivity associated with FM Co spin-clusters strongly gets affected by the development of localized 4*f* moments associated with Pr spins below 20 K, and results in such unusual reduction of magnetic coercivity. Additionally, the failure of releasing the minimum required entropy *Rln*2 (for long-range magnetic ordering), implies that either the system is subjected to an exciton-like singlet-doublet or singlet-triplet CEF transition or not all the Pr-ions present in the system participate in the magnetic ordering. Such atypical observation on the verge of competing local and itinerant magnetism in ${\text {Pr}}_2 {\text {Co}}_{0.86} {\text {Si}}_{2.88}$ compound is expected to generate extensive interest in studying microscopic magnetic interactions in related materials in the context of significant recent discovery of field-induced skyrmionic phase in isostructural magnetically frustrated material ${\text {Gd}}_2 {\text {PdSi}}_3$.

## Methods

Polycrystalline Pr–Co–Si and La–Co–Si (as non-magnetic reference) samples were prepared using conventional arc-melting procedure, taking the pure ($\ge 99.9\%$) constituent elements in the proper ratio. The ingots were remelted 6 times by flipping them after every melt to promote homogeneity. The weight loss during the melting procedure was estimated to be very small ($\le 0.5\%$). The samples were then sealed in an evacuated quartz tube and annealed for 10 days at $1000\,^{\circ }\text {C}$, thereafter cooled to room temperature at a rate of $100\,^{\circ }\text {C/hr}$. Powder x-ray diffraction (XRD) measurements at room temperature and lower temperatures have been performed using 9 kW ${\text {CuK}}_\alpha$ radiation on a TTRAX-III diffractometer (M/S. Rigaku Corp., Japan). Room temperature measurement has been carried out with solid state detector while the measurements to investigate the possible structural changes with variation in temperature, has been performed in a low temperature setup having gas detector. Zero-field neutron diffraction experiments were performed at different temperatures in the ECHIDNA beamline^92^ in ANSTO, Australia. FullProf programme package^93^ was used to analyze the XRD and ND data for structural characterization. Elemental composition and phase homogeneity have been checked through scanning electron microscope (SEM) measurements in the instrument EVO 18 (M/S. Carl Zeiss, AG, Germany) and energy dispersive analysis of X-ray (EDX) measurements were performed in a commercial Element EDS system (M/S. EDAX, USA). DC magnetizations in the temperature range 2–300 K were measured for different magnetic fields (− 70 kOe $\le$ magnetic field (*H*) $\le 70 \, \text {kOe}$) using an Evercool II VSM (M/s. Quantum Design Inc., USA) and SQUID VSM (M/s. Quantum Design Inc., USA). The dc magnetization data are taken both in zero-field-cooled (ZFC) and field-cooled (FC) protocols. In ZFC protocol, the sample is first cooled from the high temperature (essentially room temperature) to the lowest achievable temperature (2 K) in the absence of any external magnetic field, following which a magnetic field of desired strength is applied and magnetic moment is measured as a function of temperature. In contrast, in the field-cooled (FC) protocol, the system is cooled in the presence of magnetic field and the magnetization is measured in heating cycle ﻿i.e. field-cooled-warming (FCW, hence after called as FC). The measurements for $M-H$ isotherms were carried out in the ZFC mode and the system was taken to paramagnetic region before starting each measurement. Zero-field heat capacity measurement was carried out in the temperature range 2–300 K, using a commercial Physical Property Measurement System (PPMS) (M/s. Quantum Design Inc., USA). $\mu$SR measurements were carried out using EMU spectrometer at the ISIS facility, Rutherford Appleton Laboratory, UK. Details of the $\mu$SR measurements have been presented in the related section of this manuscript and supplementary material.

## Supplementary Information

Supplementary Figures.
