# Supplementary material for: Complex magnetic properties associated with competing local and itinerant magnetism in Pr2Co0.86Si2.88
Source: Sci Rep. 2021 Jun 24;11:13245. doi: 10.1038/s41598-021-90751-0 (PMC8225917; doi:10.1038/s41598-021-90751-0)
Supplement: Supplementary file 1 — Supplementary Figures. [file 41598_2021_90751_MOESM1_ESM.pdf]

Complex magnetic properties associated with competing local and itinerant magnetism in $Pr_2Co_{0.86}Si_{2.88}$

Mily Kundu,[1] Santanu Pakhira,[1,2] Renu Choudhary,[2] Durga Paudyal,[2,3] N. Lakshminarasimhan,[4,5] Maxim Avdeev,[6,7] Stephen Cottrell,[8] Devashibhai Adroja,[8,9] R. Ranganathan,[1] and Chandan Mazumdar[1]

[1]Condensed Matter Physics Division, Saha Institute of Nuclear Physics, 1/AF Bidhannagar, Kolkata 700064, India
[2]Ames Laboratory-USDOE, Iowa State University, Ames, IA, 50011, USA
[3]Electrical and Computer Engineering Department, Iowa State University, Ames, Iowa, 50011, USA
[4]Electro-organic and Materials Electrochemistry Division, CSIR-Central Electrochemical Research Institute, Karaikudi 630 003, India
[5]Academy of Scientific and Innovative Research (AcSIR), Ghaziabad 201 002, India
[6]5 Australian Nuclear Science and Technology Organisation (ANSTO), New Illawarra Road, Lucas Heights, NSW 2234, Australia
[7]School of Chemistry, The University of Sydney, Sydney, New South Wales 2006, Australia
[8]ISIS Facility, STFC, Rutherford Appleton Laboratory, Chilton, Didcot, Oxfordshire OX11 0QX, United Kingdom
[9]Highly Correlated Matter Research Group, Physics Department, University of Johannesburg, PO Box 524, Auckland Park 2006, South Africa

I. SUPPLEMENTARY MATERIALS

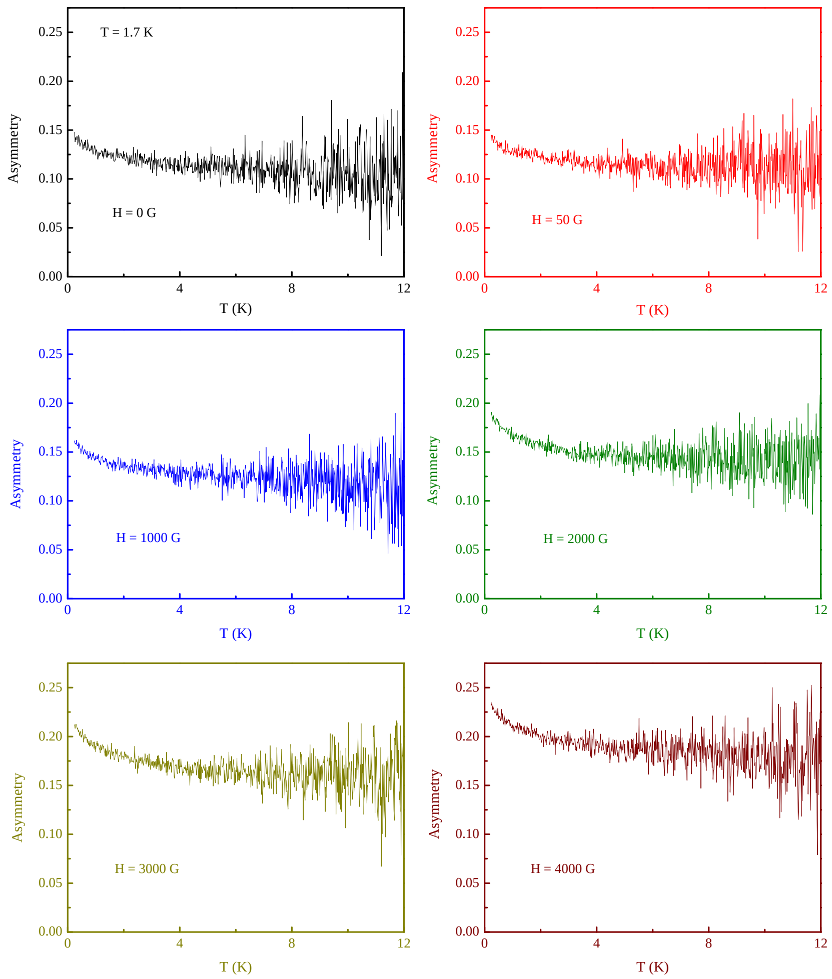

FIG. S1: Temperature dependence of asymmetry at T = 1.7 K for different fields.

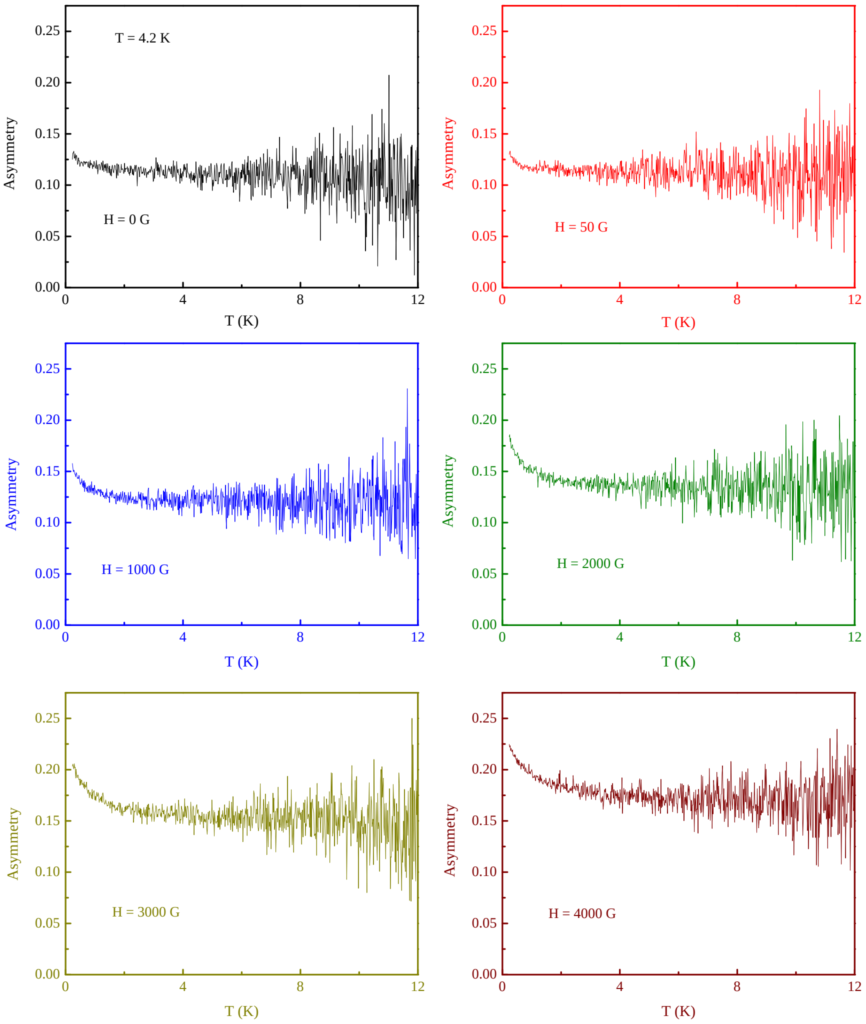

FIG. S2: Temperature dependence of asymmetry at T = 4.2 K for different fields.

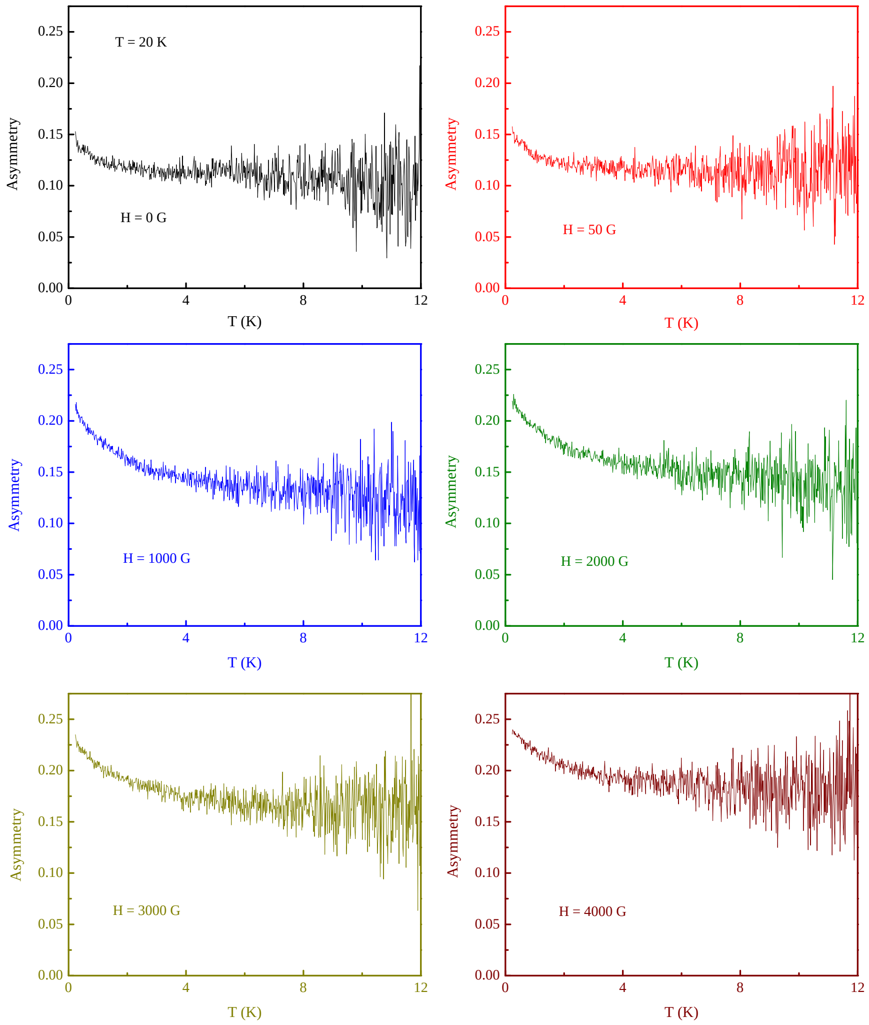

FIG. S3: Temperature dependence of asymmetry at T = 20 K for different fields.

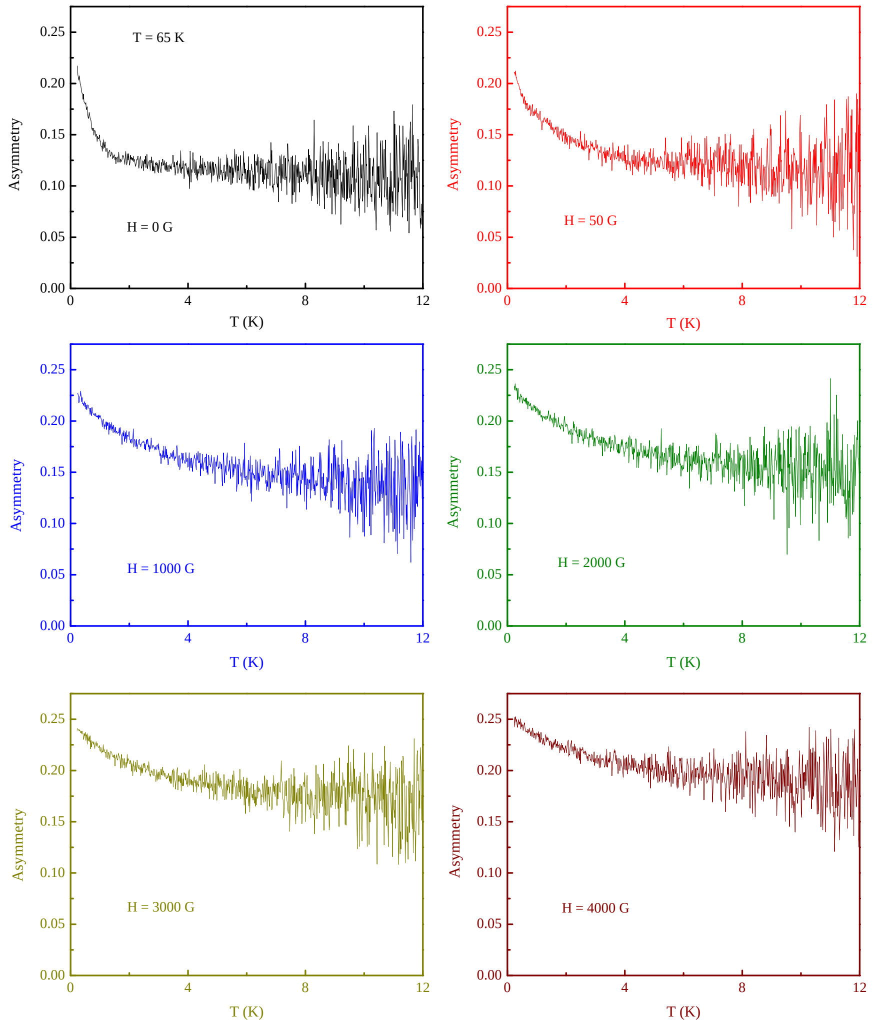

FIG. S4: Temperature dependence of asymmetry at T = 65 K for different fields.

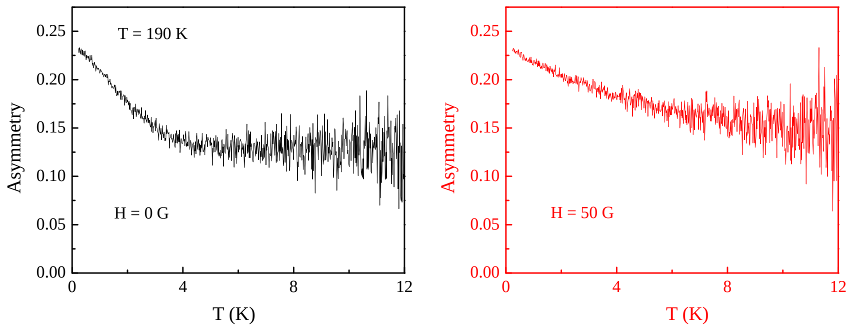

FIG. S5: Temperature dependence of asymmetry at T = 190 K for different fields.
